# Curcumin-Poly(sodium 4-styrenesulfonate) Conjugates as Potent Zika Virus Entry Inhibitors

**DOI:** 10.1021/acsami.3c13893

**Published:** 2024-01-26

**Authors:** Magdalena Obłoza, Aleksandra Milewska, Paweł Botwina, Artur Szczepański, Aneta Medaj, Piotr Bonarek, Krzysztof Szczubiałka, Krzysztof Pyrć, Maria Nowakowska

**Affiliations:** † Faculty of Chemistry, Jagiellonian University, Gronostajowa 2, 30-387 Krakow, Poland; ‡ Virogenetics Laboratory of Virology, Malopolska Centre of Biotechnology, Jagiellonian University, Gronostajowa 7a, 30-387 Krakow, Poland; § Department of Microbiology, Faculty of Biochemistry, Biophysics and Biotechnology, Jagiellonian University, Gronostajowa 7, 30-387 Krakow, Poland; ∥ Doctoral School of Exact and Natural Sciences, Jagiellonian University, Lojasiewicza 11, 30-348 Cracow, Poland; ⊥ Department of Physical Biochemistry, Faculty of Biochemistry, Biophysics and Biotechnology, Jagiellonian University, 30-387 Krakow, Poland

**Keywords:** Zika virus, antiviral, polymer, poly(sodium 4-styrenesulfonate), curcumin, flavivirus

## Abstract

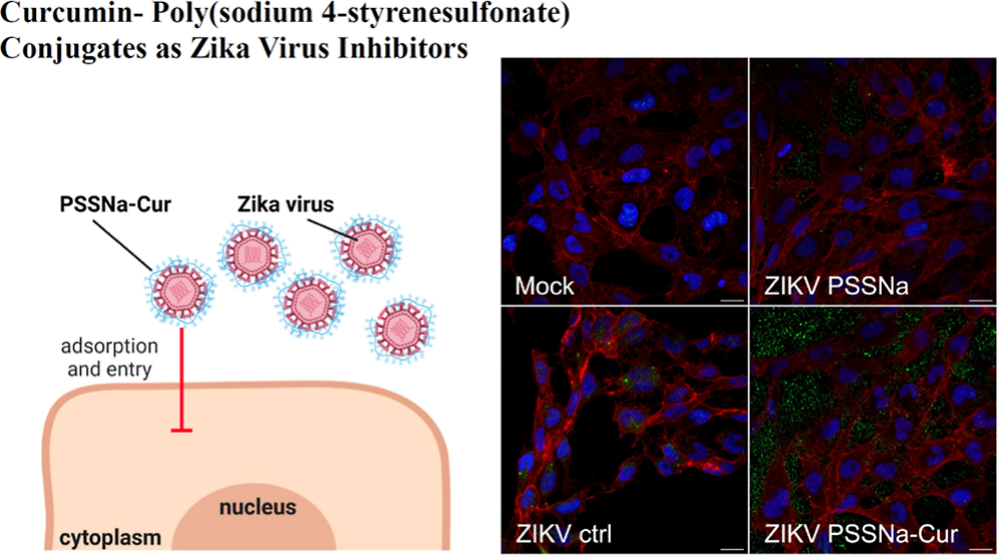

Curcumin, a natural product with recognized antiviral properties, is limited in its application largely due to its poor solubility. This study presents the synthesis of water-soluble curcumin-poly(sodium 4-styrenesulfonate) (Cur-PSSNa_*n*_) covalent conjugates. The antiflaviviral activity of conjugates was validated in vitro by using the Zika virus as a model. In the development of these water-soluble curcumin-containing derivatives, we used the macromolecules reported by us to also hamper viral infections. Mechanistic investigations indicated that the conjugates exhibited excellent stability and bioavailability. The curcumin and macromolecules in concerted action interact directly with virus particles and block their attachment to host cells, hampering the infection process.

## 1 Introduction

The Zika virus (ZIKV), a member of the Flaviviridae family, is a positive-stranded RNA virus. It was initially identified in 1947 in a rhesus monkey in the forests of Uganda.^1−3^ This mosquito-borne arbovirus is transmitted primarily by *Aedes* species but can also spread from person to person by blood or during sexual contacts.^4^ While initially the geographic distribution of the pathogen was limited to equatorial Africa, virus evolution, climate change, and increased global mobility have facilitated the spread of ZIKV worldwide, with confirmed infections reported in 91 countries across the Americas, Asia, and Europe.^5−12^ The 2016 epidemic heightened interest in ZIKV-related diseases.^12−14^ It was noticed that although ZIKV infections are often asymptomatic, in some cases, they can lead to severe neurological sequelae, including Guillain-Barré syndrome, acute inflammatory demyelinating polyneuropathy, and bilateral facial palsy.^13,14^ Importantly, ZIKV poses a significant threat to pregnant women,^15−18^ increasing the risk of miscarriage, stillbirth, and premature birth. Further, infants born to mothers who have been infected can exhibit various congenital malformations, including microcephaly, brain calcification, brain atrophy, enlarged brain chambers, paroxysmal cramps, spasticity, abnormal muscle tone, and hyperreflexia.^13^

Despite intensive global efforts, no specific treatment for ZIKV-associated diseases is currently available,^19^ which partially may be attributed to an incomplete understanding of the infection process.^20,21^ Curcumin, a natural compound with pleiotropic activity, has demonstrated broad-spectrum antiviral properties.^22−24^ Experimental data have confirmed that curcumin inhibits ZIKV in a dose-dependent manner by preventing virus attachment to the cell surface without disrupting viral RNA.^25^ Recent studies have suggested a possible additional mechanism; specifically, curcumin inhibits dengue virus, a flavivirus that is related closely to ZIKV, by allosteric binding to the NS2B-NS3 protease.^26,27^ Although curcumin shows potential as an antiviral, its effectiveness is hampered by its low solubility and instability in water.^28^ To address these challenges, different strategies have been suggested, such as the delivery of curcumin derivatives in nanosuspensions,^29^ use of drug delivery systems,^30−34^ or curcumin conjugates.^35−37^ In polymer-based conjugates, curcumin serves as the bioactive component, while the polymeric chain acts as a carrier. However, we and others have demonstrated that polymers can also function as antiviral agents^38−48^ and made an effort to include them in the new conjugates with improved antiviral activity compared to each of the components. We showed recently that poly(sodium 4-styrenesulfonate) (PSSNa) at nontoxic concentrations inhibits ZIKV replication in animal and human cells in vitro.^49^ This inhibition was also found in the pegylated variants of these polymers, i.e., in PEG-*b*-PSSNa block copolymers.^50^ Mechanistic studies indicate that PSSNa_*n*_ primarily functions through direct interaction with ZIKV particles, preventing their attachment to host cells. More precisely, the anionic PSSNa molecules are believed to engage in electrostatic interactions with the positively charged fusion loop of the ZIKV E protein dimer and the region proximal to the fusion loop.

In this study, we explored using the polymeric carrier not only as a drug delivery platform but also as an active antiviral substance. We synthesized curcumin-PSSNa (Cur-PSSNa) conjugates and observed markedly improved antiviral properties compared to PSSNa polymers of similar molecular weight. Given PSSNa’s FDA approval for hyperkalemia treatment (Kayexalate) and curcumin’s nontoxic, bioactive nature, Cur-PSSNa_*n*_ conjugates hold promise as a novel class of agents against ZIKV.

## 2 Experimental Section

### 2.1 Materials

4-Cyanopentanoic acid dithiobenzoate (CPD), 1,1′-azobis(cyclohexanecarbonitrile) (ACHN), curcumin (1,7-bis(4-hydroxy-3-methoxyphenyl)-1,6-heptadiene-3,5-dione), Cur) from *Curcuma longa*, *N*,*N′*-dicyclohexylcarbodiimide (DCC), and 4-(dimethylamino)pyridine (DMAP) were purchased from Sigma-Aldrich. Sodium styrenesulfonate (SSNa) was purchased from AK Scientific. All of the above reagents were used as received. Poly(sodium styrenesulfonate) standards (PSSNa) were purchased from American Polymer Standards. Organic solvents were purchased from POCh. Water was purified with a Millipore Milli-Q System. Dialysis tubes (MWCO = 1 and 3.5 kDa) were purchased from Spectrum Laboratories.

### 2.2 Apparatus

^1^H NMR spectra were recorded on Bruker Advance III 400 or 600 MHz in deuterated solvents. Gel Permeation Chromatography (GPC) analysis was performed at room temperature using a Right Angle Light Scattering (RALS) detector equipped with a PolySep-GFC-P Linear LC Column of 300 × 7.8 mm (Phenomenex) and a flow rate of 0.8 mL/min. A 0.1 M NaCl aqueous solution containing 20% v/v acetonitrile was used as an eluent. The molecular weights of the samples were determined using poly(sodium styrenesulfonate) (PSSNa) standards for 6 different molecular weights ranging from 2 to 140 kDa. UV–vis spectra were collected at RT in 1 cm quartz cuvettes using a single-beam photodiode array Hewlett-Packard 8452A spectrophotometer. Fluorescence spectra were collected with a HITACHI F-7100 fluorescence spectrophotometer. DLS measurements were performed with the MALVERN Zetasizer Advance Ultra. ESI-MS spectra were collected on an LC–MS 9030 mass spectrometer (Shimadzu) with electrospray ionization, quadrupole (Q), and time-of-flight (TOF) analyzer. Measurements were performed in full scan mode, i.e., without fragmentation between the Q and TOF detectors.

All ITC measurements were performed in PBS at 37 °C with a VP-ITC instrument (MicroCal, Northampton, MA, USA). All experiments were conducted in duplicate. The samples were degassed for 5 min under a vacuum prior to the measurements. In a typical procedure, 8–10 μL portions of 200 μM Cur-PSSNa_*n*_ solution were titrated as 25–30 injections into a 1435.5 μL calorimeter cell containing 20 μM HSA solution. The titrant was added every 3 min at an injection rate of 0.5 μL/s. The content of the cell was mixed at 300 rpm throughout the experiment. The data were analyzed by using MicroCal Origin software.

The circular dichroism (CD) spectra were recorded in PBS at 37 °C on a J-710 spectropolarimeter (JASCO). HSA and Cur-PSSNa_*n*_ solutions of 100 μM were used. A quartz cuvette with 10 μm or 1 cm path length was used for measurements in the far UV and near UV–vis ranges, respectively. Three to five scanning acquisitions with scanning speeds of 50 or 100 nm/min were collected. The averaged spectrum was corrected for the solvent baseline.

A Dimension Icon atomic force microscope (Bruker, Santa Barbara, CA, USA) was used to image the topography of the samples. The microscope worked in the air in the PeakForce Tapping (PFT) QNM mode, using standard silicon cantilevers (SCANASYST-AIR) with a nominal spring constant of 0.4 N/m, triangular geometry tip, and nominal tip radius of 2 nm. Cryogenic Transmission Electron Microscopy (cryo-TEM) images were collected with a Glacios Krio-TEM microscope (Thermo Fisher Scientific) at an accelerating voltage of 200 kV with a Falcon 4 Thermo Fisher Scientific detector. The volume of the samples was approximately 3 μL. They were applied on freshly glow-discharged TEM grids (Quantifoil R2/1, Cu, mesh 200) and plunge-frozen in liquid ethane with the use of a Vitrobot Mark IV (Thermo Fisher Scientific). The measurement parameters were as follows: humidity 95%, temperature 4 °C, and blot time 1 s. Frozen grids were kept in liquid nitrogen until they were clipped and loaded into the microscope.

### 2.3 Synthesis

#### 2.3.1 Curcumin-Based Chain Transfer Agent (Cur-CTA)

CPD (250 mg, 0.89 mmol), Cur (361 mg, 0.98 mmol), DCC (202 mg, 98 mmol), and DMAP (22 mg, 0.18 mmol) were dissolved in anhydrous CHCl_3_ (70 mL) in an ice bath. After 1 h, the reaction mixture was filtered to remove dicyclohexylurea, washed with saturated NH_4_Cl (50 mL) and water (3 × 50 mL), and dried with anhydrous MgSO_4_. The solvent was removed, and the crude product was purified on a silica gel column using a mixture of dichloromethane and acetone (20:1, v/v) as an eluent, affording 152 mg (27%) of Cur-CTA as an orange powder. ^**1**^**H NMR** (600 MHz, CDCl_3_): δ 15.94 (br s, 1H), 7.93 (dd, *J* = 8.5, 1.2 Hz, 2H), 7.70–7.59 (m, 3H), 7.41 (t, *J* = 7.9 Hz, 2H), 7.19–7.02 (m, 5H), 6.94 (d, *J* = 8.2 Hz, 1H), 6.55 (d, *J* = 15.8 Hz, 1H), 6.49 (d, *J* = 15.8 Hz, 1H), 5.90 (br s, 1H) 5.83 (s, 1H), 3.95 (s, 3H), 3.87 (s, 3H), 3.04–2.91 (m, 2H), 2.84–2.48 (m, 2H), 2.00 (s, 3H) ppm. ^**13**^**C NMR** (75 MHz, CDCl_3_): δ 222.4, 184.7, 181.8, 169.7, 151.4, 148.2, 147.0, 144.6, 141.3, 141.0, 139.3, 134.5, 133.2, 129.3, 128.7, 127.7, 126.8, 124.6, 123.2, 122.1, 121.9, 121.1, 118.6, 115.0, 111.6, 109.8, 101.7, 56.1, 56.0, 45.9, 33.4, 31.0, 29.7, 24.3 ppm.

#### 2.3.2 Preparation of Cur-PSSNa_*n*_ Conjugates

Conjugates of Cur-PSSNa_*n*_ were synthesized using the reversible addition–fragmentation chain-transfer (RAFT) polymerization method. The initial concentrations of SSNa, Cur-CTA (synthesized as described above), and ACHN in the reaction mixture are shown in Table 1.

**Table 1:** Polymerization Conditions for Cur-PSSNa_*n*_

	concentration		
conjugates	[SSNa] (M)	[Cur-CTA] (mM)	[ACHN] (mM)
**Cur-PSSNa**_**12**_	1	33	6.7
**Cur-PSSNa**_**26**_	1	17	3.3
**Cur-PSSNa**_**40**_	1	10	2.0

An example of the synthesis process for the conjugate is as follows: SSNa (0.5 g, 2.4 mmol), Cur-CTA (51 mg, 81.0 μmol), and ACHN (3.9 mg, 16.1 μmol) were combined in a Schlenk flask with a 1,4-dioxane-water mixture (2.5 mL, 2:3, v/v ratio). Oxygen was removed from the solution by flushing with argon for 30 min. The polymerization process was conducted at 90 °C for 4 h and terminated with air. A sample of the reaction mixture was analyzed by using ^1^H NMR to estimate monomer conversion. The conjugate was purified with dialysis in 1 or 3.5 kDa MWCO dialysis tubing against Milli-Q water and recovered by freeze-drying. The *M*_n_ and *M*_n_/*M*_w_ values were determined using GPC measurements.

To achieve a polymerization degree of 12 PSSNa chains (Cur-PSSNa_12_), the reaction was performed with a 1:5:150 initiator: Cur-CTA: monomer molar concentration ratio. That ratio was changed to 1:5:300 and 1:5:500 to obtain Cur-PSSNa_26_ and Cur-PSSNa_40_, respectively. To confirm the presence of curcumin in the α-end group, the ESI mass spectrum was collected in positive mode, and molecular ions of curcumin cations, as H^+^ and Na^+^ adducts, were observed. **Cur-PSSNa**_**12**_: [M_Cur_ + H^+^]^+^ calcd, 369.1333, observed: 369,1345, [M_Cur_ + Na^+^]^+^, calcd, 391.1152, observed: 391,1165. **Cur-PSSNa**_**26**_: [M_Cur_ + H^+^]^+^ calcd, 369.1333, observed 369,1324, [M_Cur_ + Na^+^]^+^ calcd, 391.1152, observed 391,1143. **Cur-PSSNa**_**40**_: [M_Cur_ + H^+^]^+^ calcd, 369.1333, observed 369,1325, [M_Cur_ + Na^+^]^+^, calcd, 391.1152, observed 391,1145.

### 2.4 Cells and Viruses

Vero cells (*Cercopithecus aethiops* kidney epithelial, ATCC CCL-81) and U251 cells (Human Glioblastoma, ECACC 09063001) were cultured in Dulbecco-modified Eagle’s medium (DMEM, high glucose, Life Technologies) enriched with 10% heat-inactivated fetal bovine serum (FBS, Life Technologies), along with penicillin (100 U/mL) and streptomycin (100 μg/mL), at 37 °C in a 5% CO_2_ atmosphere.

The Zika virus strain H/PF/2013 was purchased from BEI Resources. ZIKV stocks were produced by infecting Vero cells at 90% confluence with a TCID_50_ of 3000/mL. The cytopathic effect (CPE) was inspected 3 days after infection. The cells were subjected to three freeze–thaw cycles, following which supernatants were collected, aliquoted, and stored at −80 °C. The TCID_50_ of this stock was determined using the Reed and Muench method.^51^ Concurrently, a mock sample using noninfected cells was also prepared.

#### 2.4.1 Cell Viability

To assess the cytotoxicity of the compounds being studied, the XTT Cell Viability Assay kit (Biological Industries, USA) was utilized as per the manufacturer’s guidelines. In brief, Vero cells were treated with varying concentrations of the conjugates under study for 3 days at 37 °C. Postincubation, the medium was discarded, and 100 μL of new medium was added to the cells. Subsequently, 50 μL of the activated XTT solution (2,3-bis(2-methoxy-4-nitro-5-sulphenyl)-(*2H*)-tetrazolium-5-carboxanilide) was added, followed by a further incubation for 2 h at 37 °C. Absorbance was measured at a wavelength of 450 nm using a Spectra MAX 250 spectrophotometer (Molecular Devices, USA). The results were expressed as a percentage, calculated as the ratio of the signal from the tested sample to that from the control sample (cells treated with solvent) multiplied by 100%.

#### 2.4.2 RNA Isolation and RT-qPCR

The isolation of viral RNA was performed automatically using the MagnifiQ 96 Pathogen instant kit (A&A Biotechnology, Poland) and the KingFisher Flex System (Thermo Fisher Scientific, Poland), following the manufacturer’s protocol. The isolated RNA was then processed through reverse transcription (RT) and quantitative real-time PCR (RT-qPCR) using the GoTaq Probe 1-Step RT-qPCR System Protocol kit (Promega, USA). Given the potential impact of highly charged polymers on the RNA isolation process, the supernatants were diluted 1000 times before isolation.

The one-step RT-qPCR reaction used 3 μL of the isolated viral RNA in a 10 μL reaction volume. This reaction mix contained 1 × GoScript TM RT Mix for 1-Step RT-qPCR, 1 × GoTaq Probe qPCR Master Mix with dUTP, a 300 nM specific probe labeled with FAM and TAMRA (5′-FAM–CGGCATACAGCATCAGGTGCATAGGAG–TAMRA-3′), and 450 nM of each primer (5′-TTGGTCATGATACTGCTGATTGC-3′ and 5′-CCTTCCACAAAGTCCCTATTGC-3′). The process was performed in a thermal cycler (CFX96 Touch Real-Time PCR Detection System, Bio-Rad) with the following conditions: 45 °C for 15 min for reverse transcription, 95 °C for 2 min, followed by 40 cycles of 15 s at 95 °C and 30 s at 60 °C. Appropriate standards were used to determine the number of viral RNA molecules in the sample.

#### 2.4.3 Virus Inhibition

Vero or U251 cells were exposed to 100 μL of ZIKV (TCID_50_ = 2000/mL) along with 100 μg/mL of the examined conjugates/polymers. Two hours after the infection, the cells were washed three times with PBS and then incubated with the compounds under test for 4 days at 37 °C. Following this incubation, supernatants were collected, and the quantity of ZIKV RNA copies was determined using RT-qPCR.

#### 2.4.4 Immunostaining and Confocal Imaging

Cells were fixed and permeabilized using 0.1% Triton X-100 in PBS and then incubated overnight at 4 °C in PBS with an addition of 5% bovine serum albumin (BSA) and 0.5% Tween 20. For Zika virus particle visualization, the cells underwent a 2 h incubation at room temperature with rabbit anti-ZIKV E IgG (Gene Tex, cat no: GTX133314) at a 1:200 dilution. This was followed by a 1 h incubation with Alexa Fluor 488-labeled goat antirabbit IgG (Thermo Fisher Scientific, Poland) at a concentration of 2.5 mg/mL. Actin filaments were stained using Alexa Fluor 546-labeled phalloidin (Thermo Fisher Scientific, Poland) at 33 nM, and nuclear DNA was stained with 0.1 mg/mL DAPI (Sigma-Aldrich, Poland). The immunostained cultures were then mounted on glass slides using ProLong Gold antifade medium (Thermo Fisher Scientific, Poland). Fluorescent images were captured using a Leica TCS SP5 II confocal microscope (Leica Microsystems GmbH, Mannheim, Germany) and a Zeiss LSM 710 confocal microscope (Carl Zeiss Microscopy GmbH), with image acquisition and processing done using Leica Application Suite Advanced Fluorescence LAS AF v. 2.2.1 software (Leica Microsystems CMS GmbH) or ZEN 2012 SP1 software (Carl Zeiss Microscopy GmbH). Data were deconvoluted with the Huygens Essential package version 4.4 (Scientific Volume Imaging B.V., The Netherlands) and processed with ImageJ 1.47v (National Institutes of Health, Bethesda, MD, USA).

### 2.5 Statistics

All experiments were performed in at least three replicates, with each sample analyzed in triplicate. Results are presented as means ± standard deviations (SD). The 50% inhibitory concentration (IC_50_) values were calculated using Graph Pad Prism 7.0. Student’s *t*-test was employed to assess the significance of the results, with P values < 0.05 deemed significant.

## 3 Results and Discussion

### 3.1 Synthesis and Characterization of Cur-PSSNa_*n*_ Conjugates

**Scheme 1:**
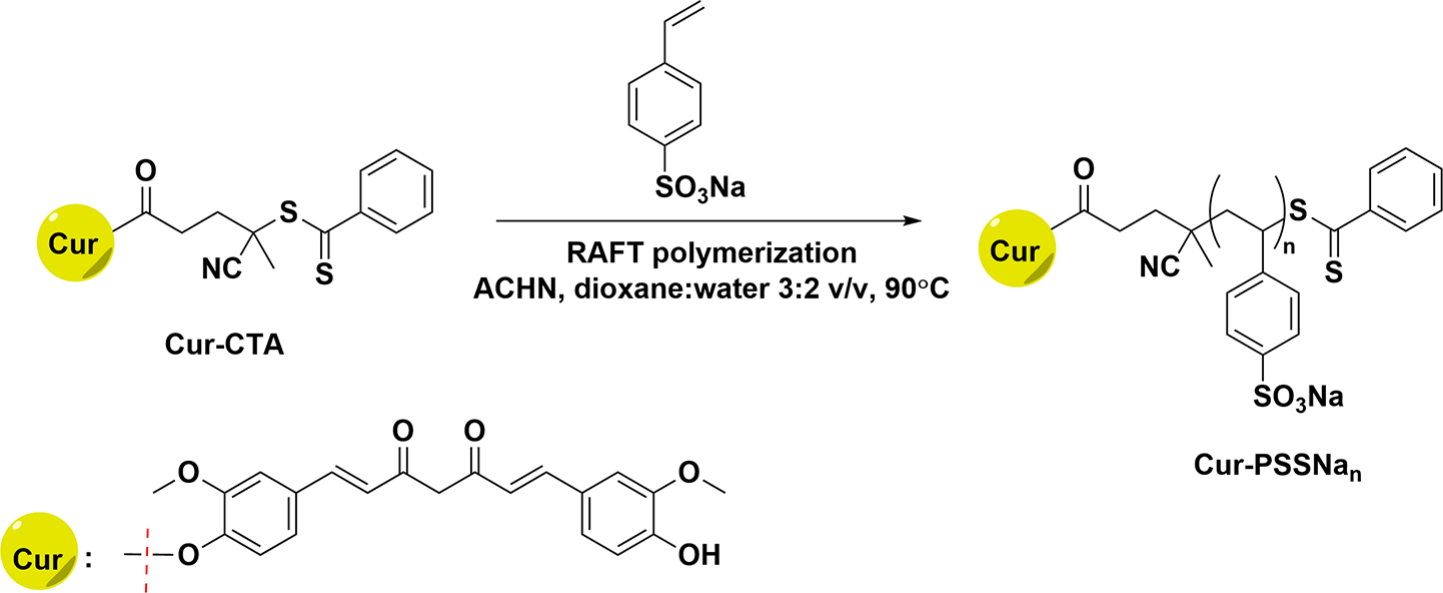
Synthesis of Cur-PSSNa_*n*_ Conjugates

The curcumin-based chain transfer agent (Cur-CTA) was prepared by reacting 4-cyanopentanoic acid dithiobenzoate (CPD) with curcumin in the presence of the DCC/DMAP coupling system. Cur-CTA was next used in the reversible addition–fragmentation chain-transfer (RAFT) polymerization of sodium 4-styrenesulfonate (SSNa) using 1,1′-azobis(cyclohexanecarbonitrile) (ACHN) as a thermal initiator in the mixture of dioxane and water (3:2, v/v), and the reaction mixture was incubated at 90 °C. These conditions allowed controlled polymerization and the generation of well-defined sulfonated oligomers/polymers with one Cur molecule at the α-end of the polymer chain (Scheme 1). The conversion of the monomer was estimated from ^1^H NMR measurements after 4 h of polymerization and reached about 50%.

The formation of Cur-based CTA was confirmed by ^1^H and ^13^C NMR spectra (Figures S1 and S2). Cur-PSSNa_*n*_ conjugates were characterized with ^1^H NMR (Figures S3 and S4), GPC (Figure S5), ESI mass spectra (Figures S6–S9), and UV–vis and fluorescence emission spectra (Figure S10) in both water (H_2_O or D_2_O) and the DMSO/water mixture. The results confirmed the formation of Cur-PSSNa_*n*_ conjugates. The ^1^H NMR spectrum (Figures S3 and S4) supported the formation of the conjugates and their micellization with Cur moieties forming the viscous hydrophobic cores of the micelles, as in D_2_O solution (a selective solvent), signals of protons from SSNa moieties only are observed (Figure S3). It features broad signals in the aromatic proton region (δ = 6–8 ppm) originating from the phenyl SSNa protons and the main chain protons (δ = 1–2 ppm). In a good solvent, signals from Cur protons also appear (Figure S4). The most intense ones (at δ = 6–8 ppm) overlap with those of aromatic PSSNa protons. The increase in the length of the polymer chain is confirmed by the decrease of the intensity of signals from Cur.

The molecular characteristics of Cur-PSSNa_*n*_ conjugates are summarized in Table 2. To calculate the theoretical number-average molecular weight, *M*_n_(theor), the following equation was used
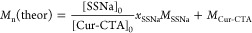
1where [SSNa]_0_ is the initial monomer concentration (mol/L), [Cur-CTA]_0_ is the initial Cur-CTA concentration (mol/L), *x*_SSNa_ is the conversion of monomer, *M*_SSNa_ is the molecular weight of SSNa (g/mol), and *M*_Cur-CTA_ is the molecular weight of Cur-CTA (g/mol). The values of *M*_n_(GPC) were close to the theoretical *M*_n_(theor) ones, and the dispersity index was low (DI = 1.1–1.3), confirming the controlled mechanism of the polymerization.

**Table 2:** Number- and Weight-Average Molecular Weight (*M*_n_ and *M*_w_, Respectively), Dispersity Index (DI), Weight Fraction of Cur in the Polymers (*w*_Cur_), and the Degree of Polymerization (DP) of Cur-PSSNa_*n*_ Conjugates

conjugate	*M*_n_(theor)^a^	*M*_n_^b^	*M*_w_^b^	*w*_Cur_	DI	DP^c^
**Cur-PSSNa**_**12**_	3720	3170	3620^b^	0.100	1.14	12
**Cur-PSSNa**_**26**_	6820	5900	7140	0.052	1.21	26
**Cur-PSSNa**_**40**_	10,940	8930	11,300	0.033	1.26	40

a Calculated from eq 1 with an estimated monomer conversion of about 50%.

b Estimated from GPC (RALS response).

c Calculated from DP = (*M*_n_ – *M*_Cur-CTA_)/*M*_SSNa_, where *M*_Cur-CTA_ and *M*_SSNa_ are molecular weights of Cur-CTA and SSNa, respectively.

Electrospray ionization (ESI) of Cur-PSSNa_*n*_ conjugates confirmed the structure of the conjugates. Curcumin cations as adducts with H^+^ and Na^+^ ions in the positive mode were efficiently produced (Figures S6–S8). What is more, fragmentation of the polymeric chain is also observed as a loss of the SSNa repeating unit or repeating unit and –CH_2_– group (−206 or −220 Da, respectively). The UV–vis absorption spectra of Cur-PSSNa_*n*_ in PBS and in the DMSO/PBS 4:1 v/v mixture revealed a weak band with a maximum around 430 nm, characteristic of Cur (Figure S10A,B). The comparison of the UV–vis spectra of the conjugates reveals a strong hypochromic effect of increasing chain length, which could be ascribed to interactions of the π electrons of curcumin with the polymeric chains, similar to those observed in the curcumin-DNA system.^52^ A weak fluorescence of the Cur moiety in the conjugates with an emission maximum of about 525 nm can be observed upon excitation with a wavelength of λ_ex_ = 420 nm (Figure S10C,D).

**Figure 1:**
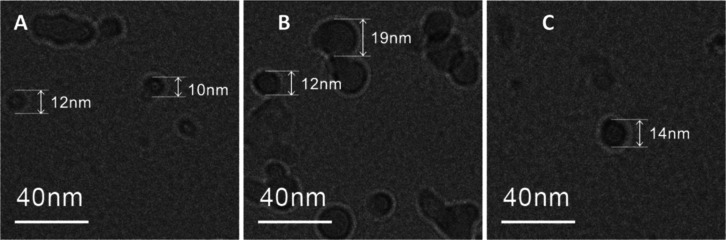
Cryo-TEM images of Cur-PSSNa_12_ (A), Cur-PSSNa_26_ (B), and Cur-PSSNa_40_ (C) conjugates (1 mg/mL) in PBS.

The cryo-TEM and AFM images revealed the formation of spherical droplet-like aggregates of Cur-PSSNa_*n*_ with 10–25 nm diameters, suggesting micelle formation (Figure 1, Table 3, Figure S11). They are negatively charged, with zeta potential values ranging from −21 to −24 mV (Table 3).

**Table 3:** Values of Micelles Diameter (*D*_cryo_, 1 mg/mL, D_AFM_, 0.01 mg/mL), Zeta Potentials, ζ, for Cur-PSSNa_*n*_ Conjugates (*c* = 3.3 × 10^–8^ M in PBS, pH = 7.4, *T* = 37 °C), and Critical Micellization Concentration (CMC)

conjugate	*D*_cryo_ (nm)	*D*_AFM_ (nm)	zeta potential, ζ (mV)	CMC (mM)
**Cur-PSSNa**_**12**_	10	25.2 ± 9.2	–21.1 ± 2.7	0.049
**Cur-PSSNa**_**26**_	12	23.7 ± 7.1	–24.0 ± 1.7	0.047
**Cur-PSSNa**_**40**_	14	22.2 ± 8.4	–22.7 ± 0.7	0.044

The critical micellization concentration of Cur-PSSNa_*n*_ was determined using diphenyl-1,3,5-hexatriene (DPH) as a fluorescence probe. It was equal to 0.049, 0.047, and 0.049 mM for Cur-PSSNa_12_, Cur-PSSNa_26_, and Cur-PSSNa_40_, respectively (Table 3, Figure S12).

### 3.2 Cytotoxicity of Cur-PSSNa_*n*_ Conjugates

**Figure 2:**
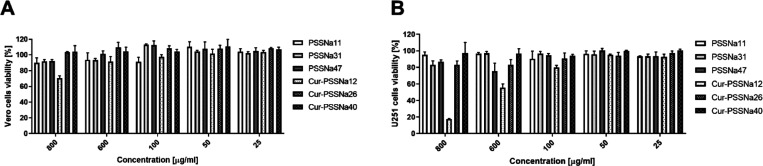
Comparative cytotoxicity of Cur-PSSNa_*n*_ and corresponding PSSNa_*n*_ controls of various molecular weights in Vero (A) and U251 cells (B). The presented data are normalized as a percentage of viable cells relative to samples treated with the solvent (DMSO). All experiments were conducted in triplicate, and average values with standard deviations (error bars) are presented.

The cytotoxicity of the Cur-PSSNa_*n*_ and PSSNa_*n*_ polymers (used as a reference) was examined using an XTT assay. Confluent Vero and U251 cells were incubated for 3 days in media supplemented with conjugates with differing polymer chain lengths at concentrations of 25–500 μg/mL. As shown in Figure 2 and Table 4, the conjugates were characterized by low toxicity; however, at the highest concentration of the conjugates tested, i.e., 500 μg/mL, the conjugate with the shortest polymeric chain (Cur-PSSNa_12_) was more toxic than the reference polymer (∼75% viability compared with the reference PSSNa_11_) and also more toxic than the conjugates with longer polymeric chains. That can be explained considering that under these conditions, the Cur concentration equals 136 μM, considerably exceeding its toxic concentration (vide infra).

**Table 4:** Cytotoxicity of Polymers. The 50% Cellular Cytotoxicity (CC_50_) Values Were Determined in Vero and U251 Cells

compound	cellular cytotoxicity 50% (CC_50_) [mg/mL]	
	Vero cells	U251 cells
PSSNa_11_	8.11 (2.91 mM)	4.16 (1.50 mM)
PSSNa_31_	4.49 (0.81 mM)	1.92 (0.35 mM)
PSSNa_47_	5.54 (0.67 mM)	1.87 (0.22 mM)
Cur-PSSNa_12_	0.93 (0.29 mM)	0.69 (0.22 mM)
Cur-PSSNa_26_	1.65 (0.28 mM)	1.22 (0.21 mM)
Cur-PSSNa_40_	2.38 (0.26 mM)	1.86 (0.20 mM)

For comparison, the cytotoxicity of Cur originally dissolved in DMSO was also examined using the same cell lines. The XTT assay revealed significant toxicity of 40 μM Cur in both cell types (Figure S13). Some toxicity (∼30–40%) and morphological changes in the cells (data not presented) were observed at 20 μM Cur, whereas 10 μM was the highest nontoxic concentration for both cell lines.

### 3.3 Inhibition of ZIKV Replication by Cur-PSSNa_*n*_ Conjugates

**Figure 3:**
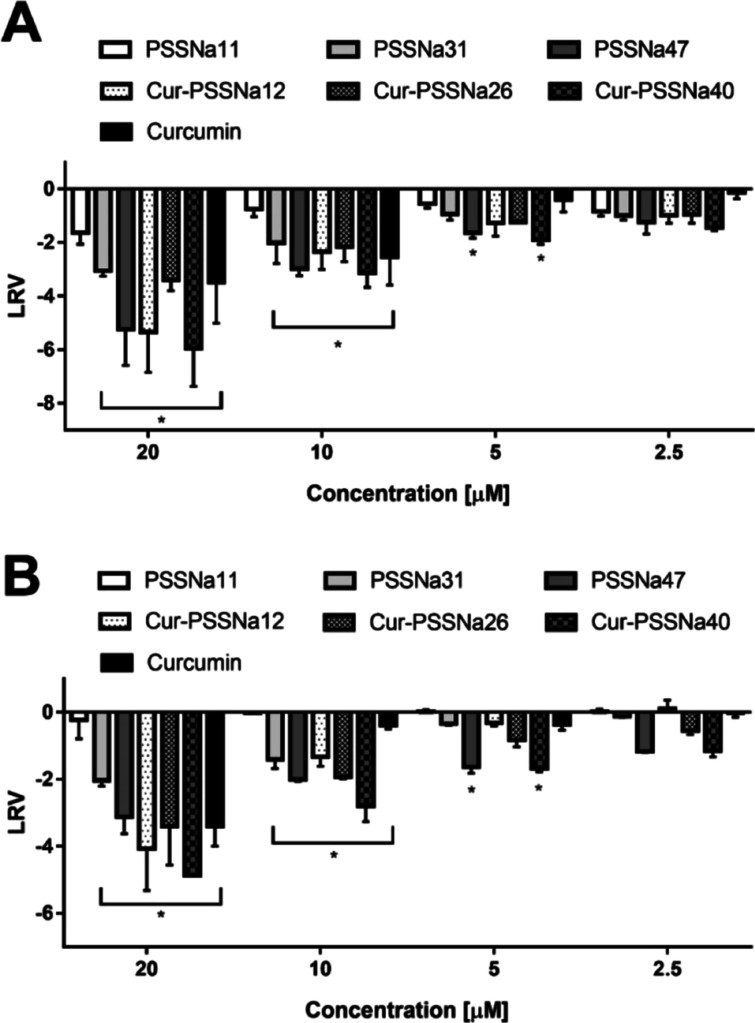
Suppression of ZIKV replication in Vero (A) and U251 (B) cells by Cur-PSSNa_*n*_ conjugates, PSSNa_*n*_ polymers, and curcumin was assessed through quantitative RT-qPCR. Results are shown as the log removal value (LRV) relative to untreated samples (DMSO control). This experiment was conducted with six biological replicates, and the data are displayed as average values accompanied by standard errors. Statistical significance was noted where *P < 0.05.

For assessing the effectiveness of Cur-PSSNa_*n*_ conjugates against ZIKV, Vero and U251 cells were exposed to the virus along with either the conjugates or PSSNa_*n*_ polymers at concentrations varying between 2.5 and 20 μM, significantly lower than the levels deemed toxic. Curcumin was used at the same molar concentrations as in the conjugated version. In both cell lines, ZIKV infection was significantly inhibited by all tested compounds, an effect that has been reported previously for PSSNa_*n*_ polymers^49^ and Cur alone (Figures 3, S14 and Table 5).^25^ The number of ZIKV particles/copies formed in the presence of Cur-PSSNa_*n*_ conjugates was considerably lower (by up to 6 orders of magnitude) than the number formed in the negative control samples (PBS). In addition, conjugates with longer PSSNa chains exhibited a stronger inhibitory effect in comparison to shorter conjugates. At suboptimal concentrations (5–10 μM), the antiviral effect of Cur-PSSNa_*n*_ conjugates was markedly higher than that of PSSNa_*n*_ polymers or Cur alone.

**Table 5:** Inhibitory Concentration IC_50_ Values Determined in Both Cell Types

compound	inhibitory concentration 50% (IC_50_) [μM]	
	Vero cells	U251 cells
PSSNa_11_	11.08	10.35
PSSNa_31_	2.36	3.43
PSSNa_47_	1.26	1.87
Cur-PSSNa_12_	2.88	3.74
Cur-PSSNa_26_	2.06	2.16
Cur-PSSNa_40_	1.57	1.63

### 3.4 Mechanism Underlying the Anti-ZIKV Activity of Cur-PSSNa_*n*_ Conjugates

**Figure 4:**
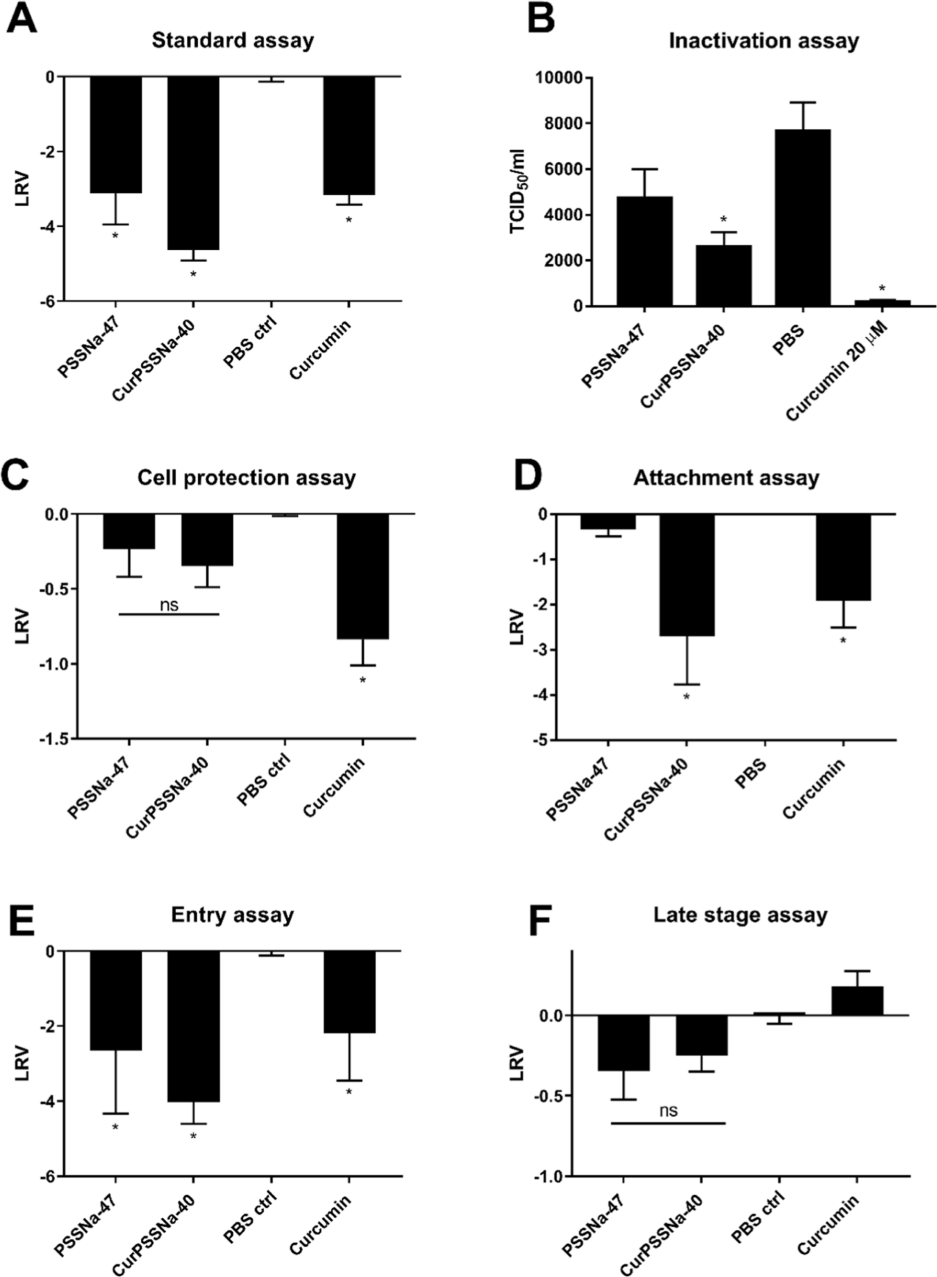
Functional assays for determining the Cur-PSSNa_*n*_ mechanism of action. Virus replication was evaluated using quantitative RT-qPCR. The data are presented as log removal value [LRV, panels: (A,C,D,E,F)] compared to untreated samples (negative medium control) or as virus titer [TCID_50_/mL] (B). The assay was performed in triplicate, and average values with standard errors are presented. *P < 0.05.

Having the antiviral effect well characterized, we performed functional assays using the conjugate with the longest PSSNa chains, the corresponding PSSNa polymer, Cur alone, and phosphate-buffered saline (PBS) as a negative control. In the first experiment (“standard assay”), 20 μM Cur-PSSNa_40_, PSSNa_47_, Cur, or PBS was present during all stages of virus infection (Figure 4A). In this assay, ZIKV infection of U251 cells was inhibited significantly by Cur-PSSNa_40_, PSSNa_47_, and Cur. A subsequent “inactivation assay” was performed to examine the direct inactivation of the virus by the test compounds. In this assay, virions were incubated with 20 μM Cur-PSSNa_40_, PSSNa_47_, Cur, or PBS for 6 h at room temperature with mixing. After preincubation, the samples were titrated on confluent Vero cells. The assay showed a decline in virus infectivity after preincubation with the tested samples compared to the negative control (PBS) (Figure 4B). This result agrees with those of our previous study and the literature data showing the direct inactivation of enveloped viruses such as ZIKV by Cur.^25^

We conducted a “cell protection assay” to evaluate whether the test compounds could interact with host cells and shield them from infection. U251 cells were treated with 20 μM of Cur-PSSNa_40_, PSSNa_47_, curcumin, or in PBS media for 1 h at 37 °C. Post-treatment, the cells were washed thrice, with PBS and then infected with ZIKV (TCID_50_ = 2000/mL). Following a 4 day incubation at 37 °C, supernatants were collected, and the quantity of ZIKV RNA copies was measured using RT-qPCR. The findings indicated that none of the test compounds significantly hindered virus infection when compared to the negative control (PBS) (Figure 4C). An “attachment assay” was conducted to assess if the test compounds could prevent virus particles from attaching to host cells. For this purpose, U251 cells were first chilled at 4 °C and then exposed to ZIKV (TCID_50_ = 4000/mL) along with 100 μL of 20 μM Cur-PSSNa_40_, PSSNa_47_, curcumin, or PBS. The cells were maintained at 4 °C for 3 h, allowing virus attachment but not entry into the host cells, followed by three washes with PBS. Afterward, 100 μL of fresh medium was added, and the cells were incubated for 4 days at 37 °C. Postincubation, supernatants were collected for RT-qPCR analysis. The outcomes revealed a notable reduction in ZIKV infection in cells treated with either the Cur-PSSNa conjugates or curcumin alone compared to the negative control (PBS) (Figure 4D). We carried out an “entry assay” to determine if the test compounds impeded the internalization of the virus into cells. Initially, U251 cells were cooled and inoculated with ice-cold ZIKV (TCID_50_ = 2000/mL) and then held at 4 °C for 2 h to facilitate virus binding. Afterward, the virus particles were removed by washing with ice-cold PBS, and the cells were treated with 20 μM Cur-PSSNa_40_, PSSNa_47_, curcumin, or PBS at 37 °C for 2 h, allowing virus penetration. Following this, the medium was discarded, and the cells were washed thrice with an acidic buffer to block the ability of uninternalized virions to fuse. The effectiveness of the virus deactivation by the low pH was confirmed in prior experiments (data not shown). The cells were then rinsed with PBS, covered with culture medium, and incubated at 37 °C for 4 days. RT-qPCR analysis of the supernatants indicated that Cur-PSSNa_40_, PSSNa_47_, and curcumin significantly hindered ZIKV entry compared to the negative control (PBS) (Figure 4E). Finally, a “late-stage assay” was performed to examine whether the test compounds hampered the late stages of ZIKV replication (i.e., virus replication, assembly, and egress). In this test, U251 cells were exposed to ZIKV (TCID_50_ = 500,000/mL) and then incubated for 2 h at 37 °C, providing time for the virus to penetrate the cells. Subsequently, the cells were rinsed three times with PBS and then incubated in culture medium containing 100 μL of 20 μM Cur-PSSNa_40_, PSSNa_47_, Cur, or PBS for 24 h at 37 °C, after which the supernatants were sampled for RT-qPCR analyses. Prior to this assay, we verified that a 24 h incubation and a high virus titer (TCID_50_ = 500,000/mL) were necessary for the analysis of the single replication cycle (data not shown). The assay showed that none of the compounds affected the late stage of infection (Figure 4F).

**Figure 5:**
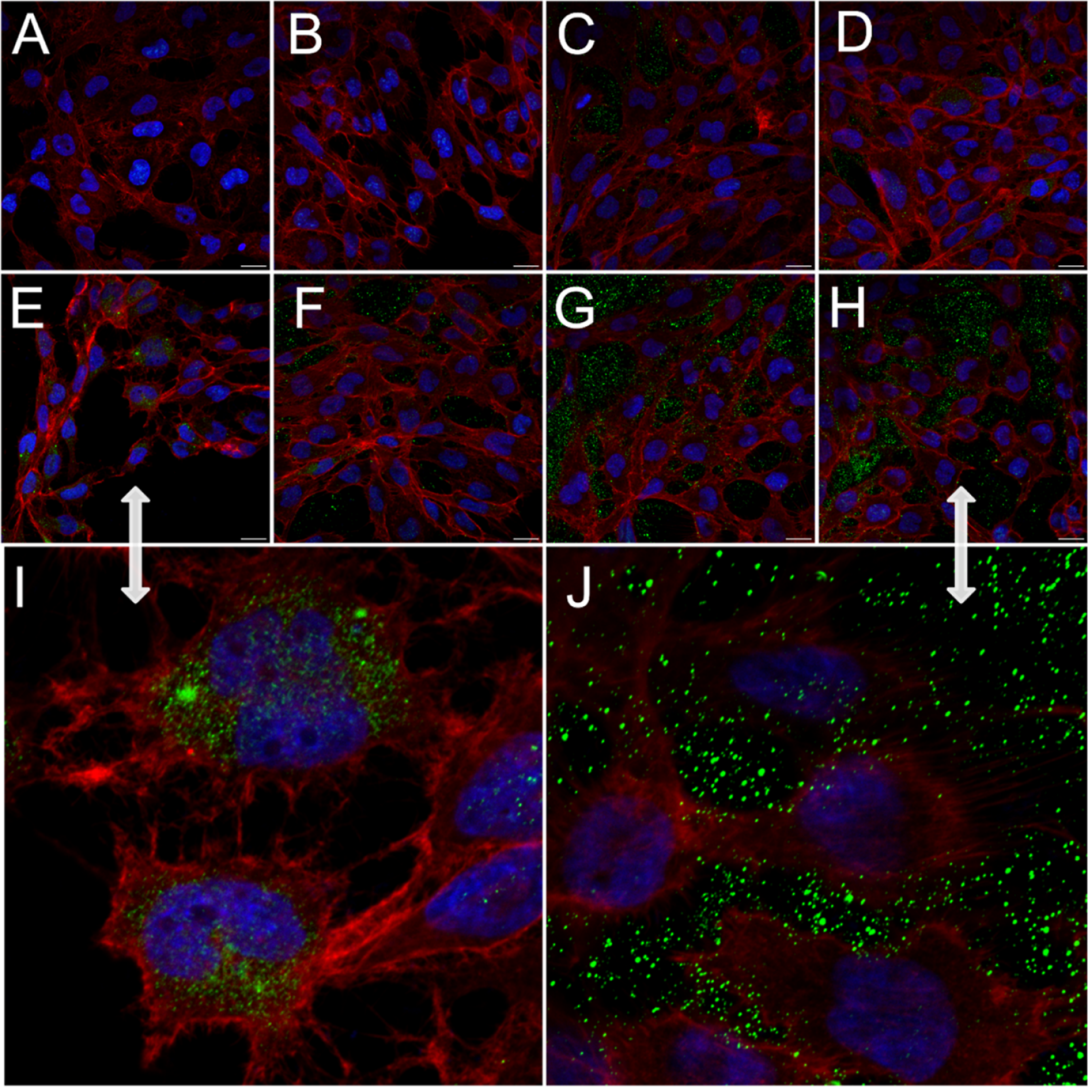
ZIKV adhesion to U251 is hampered by Cur-PSSNa_*n*_ conjugates. Cells were incubated with mock lysate (A) or virus in the presence of PSSNa_11_ (B), PSSNa_31_ (C), PSSNa_47_ (D), control PBS (E) or conjugates: Cur-PSSNa_12_ (F), Cur-PSSNa_26_ (G), or Cur-PSSNa_40_ (H). Zoomed images of control PBS and Cur-PSSNa_40_ are presented in panels (I,J), respectively. Virus E protein was stained with specific antibodies (shown in green), and actin in the filaments was stained with phalloidin (shown in red). Nuclei were stained with DAPI (shown in blue). ZIKV adhesion was analyzed with confocal microscopy. Scale bar: 20 μm (A–H).

The results described above suggested that the Cur-PSSNa_*n*_ conjugates interfered with the ability of ZIKV to attach to and enter target cells; therefore, we attempted to observe these phenomena using fluorescence microscopy. Precooled U251 cells were overlaid with ice-cold ZIKV in the presence of Cur-PSSNa_*n*_ conjugates, PSSNa_*n*_ polymers, PBS, or Cur. After incubation for 3 h at 4 °C, the cells were washed thrice with PBS, fixed with 3.7% w/v paraformaldehyde, and stained using antibodies targeting the ZIKV envelope protein. Confocal microscopy analyses of virus adhesion showed that Cur-PSSNa_*n*_ conjugates and control PSSNa_*n*_ polymers markedly inhibited the binding of ZIKV to cells. For the conjugate samples, virus particles could be observed only outside the cells, possibly as a result of the repulsive electrostatic interactions of the conjugate-coated virus with the cell membrane.^46^ By contrast, ZIKV particles were attached to control (PBS) cells, mainly on actin filaments (Figure 5).

### 3.5 Interaction of Cur-PSSNa Conjugates with Human Serum Albumin

**Figure 6:**
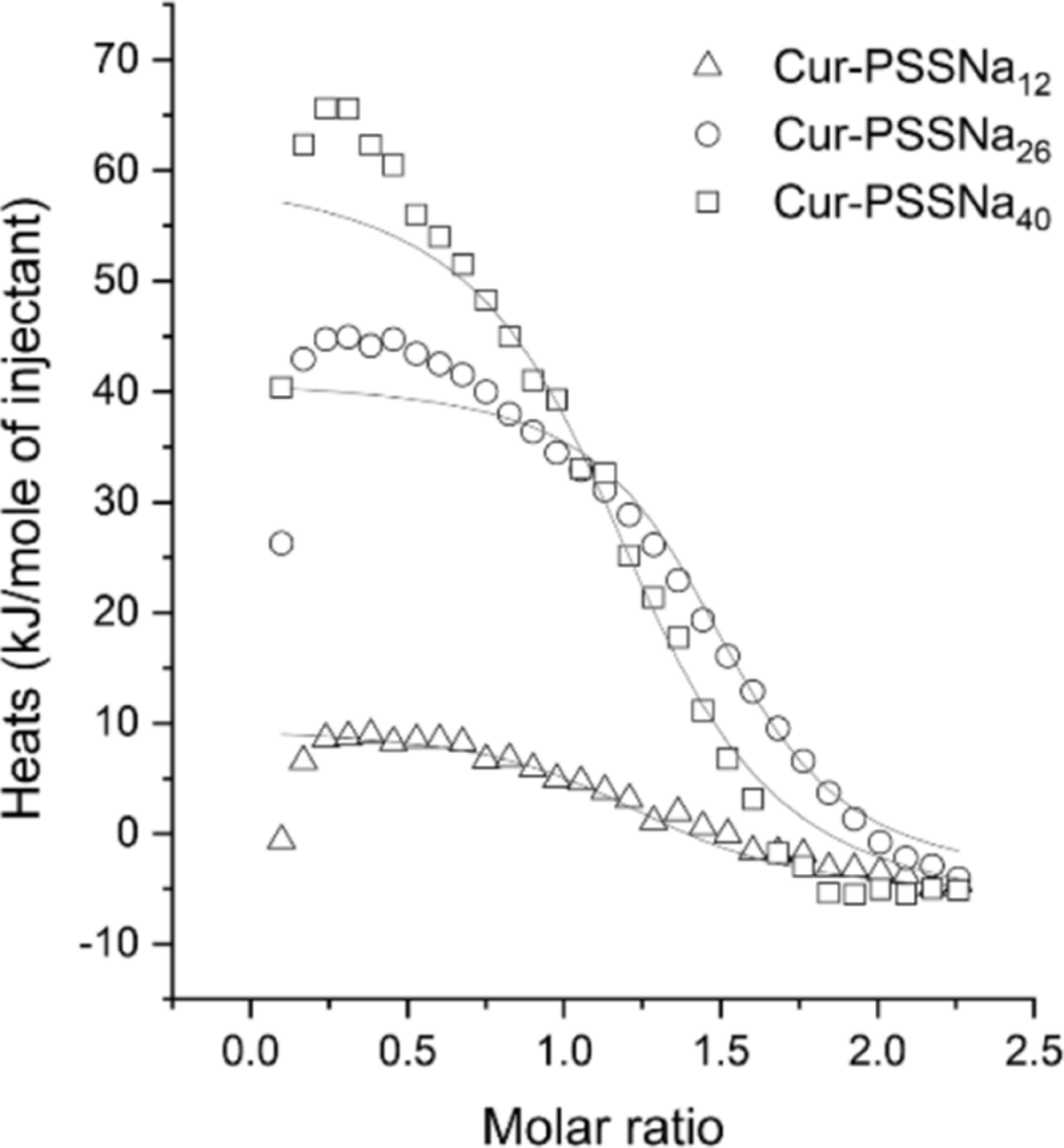
Calorimetric isotherms depicting the interaction of Cur-PSSNa_*n*_ polymers with HSA. The isotherms were measured in PBS at 37 °C. The lines shown illustrate the representative best-fit curves obtained assuming the model of a single class of binding sites.

The interaction of Cur-PSSNa_*n*_ with HSA was studied using cryogenic transmission electron microscopy (cryo-TEM), the dynamic light scattering technique (DLS), isothermal titration calorimetry (ITC), circular dichroism, and zeta potential measurement. The results of these studies (see Tables 6, 7, Figure 6, Table S1, and Figures S15–S18) indicated that the Cur-PSSNa conjugates interacted with HSA to form well-defined molecular aggregates. In buffer solution, the aggregates existed as wormlike, negatively charged nanoparticles (Figure S15) with dimensions of 7–9 nm (Table 6). The cryo-TEM images confirmed that interactions of Cur-PSSNa_*n*_ conjugates with HSA provide small structures with sizes in the 9–14 nm range (Figure S16). The values of their ζ-potential ranged from −8.8 to −12.5 mV, increasing with the length of the polymeric chain. CurPSSNa_40_ + HSA aggregates, characterized by a ζ-potential of −12.5 mV, would be expected to form a stable, electrostatically, and sterically stabilized dispersion in aqueous media. Similar results were obtained when Cur-PSSNa_*n*_ conjugates were incubated with bovine serum albumin (BSA), another model protein (Table S1, Figure S15A).

**Table 6:** **Cryo-TEM** Dimension (Both Protein and Conjugates Concentration was Equal to 1.5 × 10^–5^ mol/dm^3^) and Zeta Potential of HSA-Polymer Aggregates in PBS (Both Protein and Conjugates Concentration was Equal to 3.3 × 10^–8^ mol/dm^3^)

polymer/aggregate	*d* [nm]	zeta potential, ζ (mV)
**HSA**	8^53^	–7.5 ± 1.4
**CurPSSNa**_**12**_ + **HSA**	9	–8.8 ± 3.4
**CurPSSNa**_**26**_ + **HSA**	14	–11.0 ± 0.5
**CurPSSNa**_**40**_ + **HSA**	14	–12.5 ± 1.3

**Table 7:** Thermodynamic Parameters of the Interaction Between Cur-PSSNa_*n*_ Polymers and Human Serum Albumin (HSA)^a^

conjugate	stoichiometry	*K*_a_ (×10^6^ M^–1^)	Δ*H*_a_ (×10^4^ J/mol)	Δ*S*_a_ (×10^2^ J/mol/K)	Δ*G*_a_ (×10^4^ J/mol)	*K*_d_ (×10^–7^ M)
**Cur-PSSNa**_**12**_	0.79 ± 0.05	1.16 ± 0.57	1.9 ± 0.3	1.8 ± 0.1	–3.60 ± 0.13	8.6 ± 4.2
**Cur-PSSNa**_**26**_	0.67 ± 0.04	2.66 ± 1.43	6.8 ± 1.1	3.4 ± 0.3	–3.81 ± 0.14	3.8 ± 2.0
**Cur-PSSNa**_**40**_	0.82 ± 0.03	1.12 ± 0.36	8.2 ± 0.7	3.8 ± 0.2	–3.59 ± 0.08	8.9 ± 2.9

a *K*_a_—affinity constant, Δ*H*—enthalpy change, Δ*G*—free enthalpy change, and Δ*S*—entropy change.

### 3.6 Calorimetry Analysis of Cur-PSSNa_*n*_ + HSA Aggregates

The thermodynamics of the interaction between Cur-PSSNa_*n*_ and HSA were examined using ITC. The data are presented in Figure 6 and Table 7, and the raw data are presented in Figure S17. The process was analyzed assuming the one-class binding site model and that the ligand was a protein. The stoichiometry was close to one, suggesting complexation with a molar ratio of 1:1. The process is endothermic and entropically driven, with Δ*G*_a_ being in the range of −36 to −38 kJ/mol. A marked entropy increase was observed, which can be ascribed to the release of ions and water molecules during the polymer–protein interaction.

### 3.7 Circular Dichroism

**Figure 7:**
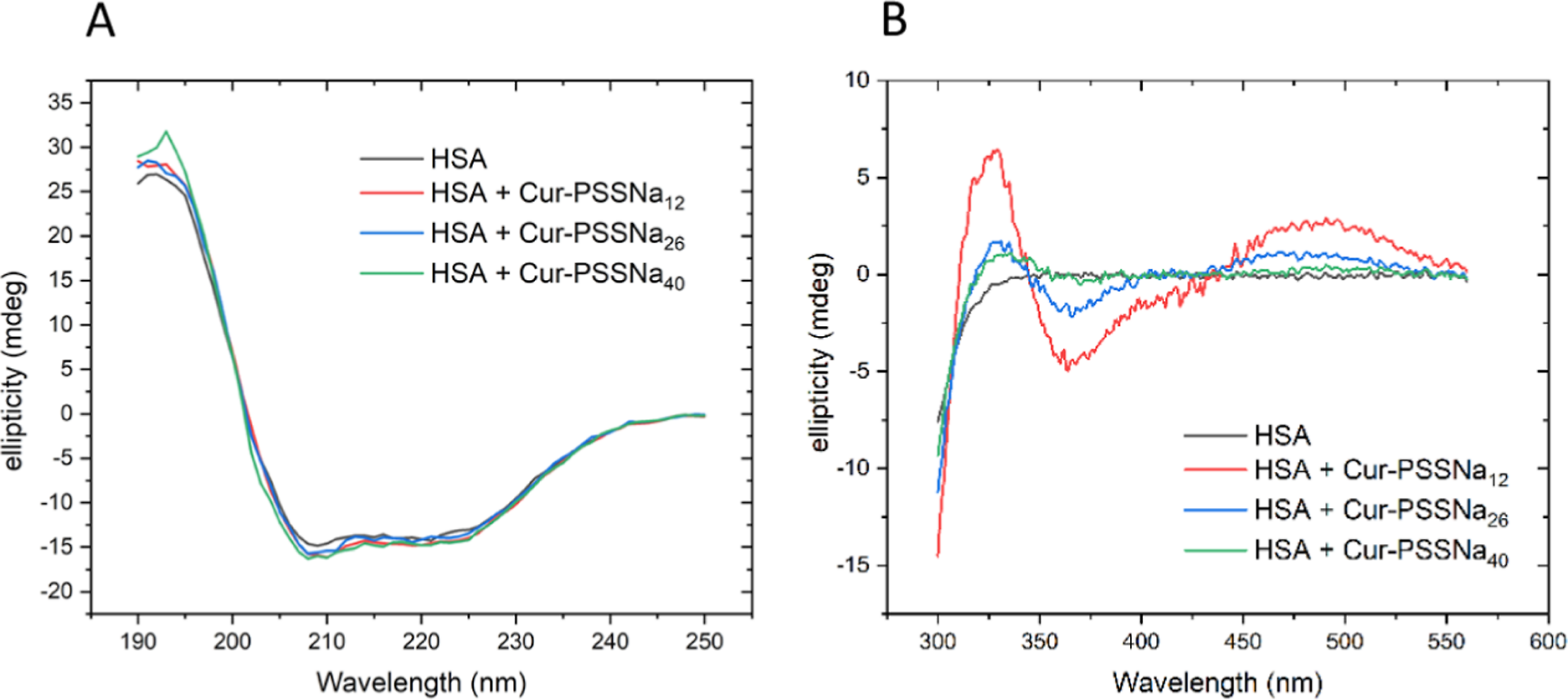
Circular dichroism spectra of HSA and HSA-Cur-PSSNa_*n*_ complexes in PBS, pH 7.4, at 37 °C. HSA and Cur-PSSNa_*n*_ solutions of 100 μM were used. (A) far UV range and (B) near UV–vis range.

In order to assess the nature of the interaction between HSA and Cur-PSSNa_*n*_ conjugates, CD measurements were performed for 100 μM concentrations of HSA and Cur-PSSNa_*n*_. Taking into account the values of association constants, one can expect that under such experimental conditions, more than 90% of the molecules are complexed. In the far UV range (190–250 nm), the spectra obtained for HSA in the absence and presence of Cur-PSSNa_*n*_ did not differ (Figure 7, left panel). This result indicates that the HSA-polymer interaction does not significantly disturb the secondary structure of the protein. In the near UV–vis range (300–600 nm, Figure 7, right panel), the CD spectra of the complexes show three extrema centered at 330, 365, and 490 nm, which are absent in the HSA spectrum. The intensity of the observed CD signal is strongest for Cur-PSSNa_12_ and decreases with an increase in PSSNa chain length. A similar CD signal was obtained earlier for HSA complexes with curcumin alone, originating from the optically active π–π* transition of the curcumin molecule surrounded by the asymmetric environment of HSA upon binding.^54^ Therefore, our result indicates that polymers interact with HSA directly through conjugated Cur. Importantly, it was observed that this interaction is beneficial as it preserves the curcumin structure and biological activity.^55,56^

The results of the experiments presented above confirmed the important role of Cur in the antiviral activity of Cur-PSSNa_*n*_ conjugates. That is in line with the previous observation of its broad antimicrobial activity.^57^ Researchers have observed a dose- and time-dependent decrease in virus yields in vitro at micromolar concentrations of Cur.^58^ CC_50_ (∼53 μM) and IC_50_ (∼5–14 μM) values have been determined for Cur in DMSO solutions in Vero E6 cells infected with various ZIKV strains.^59^ In addition, time-of-addition experiments have shown that Cur acts against ZIKV exclusively during the early stages of infection, affecting cell attachment and/or entry, without virucidal activity at later stages.^60^ It has been suggested that the ability of Cur to affect the cell attachment of ZIKV might be explained by an effect on membrane fluidity.^25^ In addition, an in silico analysis has been performed to identify the target protein(s) of Cur in the cell membrane.^61^ The molecular docking study considered four targets: TP53, AKT1, PTEN, and TNF, and found a strong interaction between Cur and TNF. Notably, previous in vitro experiments have shown dose-dependent antiviral effects of Cur at 12.5–50 μM, with no antiviral activity at concentrations lower than 10 μM.^59^

Although Cur demonstrates promising antiviral properties, concerns have been raised about its potential adverse effects. The FDA considers Cur to be generally safe in food at concentrations of 1–20 mg/100 g of food product.^60^ In vitro studies have indicated that the toxicity of Cur is dependent on various parameters, such as the concentration, duration of treatment, and type of cells used.^62^ Notably, in a previous study, human dermal fibroblasts, which are particularly permissive to ZIKV, were highly sensitive to Cur; the toxic effects were recorded already at concentrations >10 μM.^63^ In agreement with this result, we found here that 10 μM Cur was the highest nontoxic concentration for Vero and U251 cells. Although the mechanism of toxicity is not fully clear, previous experiments have shown that Cur binds to the minor groove of DNA.^63^

To overcome concerns regarding the safety of Cur as an antiviral agent, we propose the use of water-soluble Cur-PSSNa_*n*_ conjugates (the solubility of Cur in a pH 5.0 aqueous buffer is as low as 11 ng/mL^64^). The high bioavailability of these conjugates would allow the elimination of DMSO as a solvent and obtaining the antiviral effect at lower and less toxic concentrations of Cur. Further, we used an FDA-approved drug for treating hyperkalemia (PSSNa), which by itself carries antiviral activity, as a component of the conjugate.^49^ The Cur-PSSNa_*n*_ conjugates described here demonstrated significantly stronger antiviral properties than PSSNa_*n*_ polymers of similar average molecular weight.

Considering the potential practical applications of Cur-PSSNa_*n*_ conjugates and their existence as negatively charged nanoparticles in aqueous environments, we studied their interaction with the major protein of human plasma, i.e., HSA. Using DLS, cryo-TEM, and ITC techniques, we found that Cur-PSSNa_*n*_ conjugates spontaneously formed small, nanometric (approximately 10–14 nm), negatively charged complexes with HSA having 1:1 stoichiometry. The circular dichroism test indicated that Cur-PSSNa_*n*_ can interact with HSA directly through Cur. The zeta potential values of HSA-Cur-PSSNa_*n*_ nanoparticles depended on the length of the polyelectrolyte chain within the conjugate, and the complexes formed between HSA and Cur-PSSNa_26_ or Cur-PSSNa_40_ were able to form stable dispersions in the buffer solution.

## 4 Conclusions

This study describes the synthesis and antiviral properties of a series of water-soluble Cur-PSSNa_*n*_ conjugates with high bioavailability. The conjugates exhibited low cytotoxicity across a wide range of concentrations, and their antiviral effectiveness was proportional to the length of the polymer chain. Cur-PSSNa_*n*_ conjugates were more potent inhibitors of ZIKV than the polymers or Cur alone, indicating a clear benefit also from the perspective of efficacy. Our findings demonstrate that Cur-PSSNa conjugates interact directly with virus particles and block the virus attachment to host cells, hampering the infection process. The structure and biological activity of Cur in Cur-PSSNa_*n*_ conjugates can be preserved due to the formation of micellar aggregates with Cur located in their interiors and the formation of stoichiometric complexes with HSA.

## Supporting Information Available

^1^H NMR and ^13^C NMR, ESI-MS, UV–vis, fluorescence spectra of all synthesized compounds, GPC chromatograms of Cur-PSSNa_*n*_ conjugates, experimental data used for determination of cmc for conjugates, AFM images, Cur cytotoxicity in Vero and U251 cells, Cur inhibition of ZIKV replication, dimension, dispersity index, zeta potential, and cryoTEM images of conjugate-protein (HSA and BSA) aggregates, and calorimetric isotherms of the binding of Cur-PSSNa_*n*_ conjugates to HSA (PDF)
