# Supplementary material for: Curcumin-Poly(sodium 4-styrenesulfonate) Conjugates as Potent Zika Virus Entry Inhibitors
Source: ACS Appl Mater Interfaces. 2024 Jan 26;16(5):5426–37. doi: 10.1021/acsami.3c13893 (PMC10859898; doi:10.1021/acsami.3c13893)
Supplement: Supplementary file 1 — am3c13893_si_001.pdf [file am3c13893_si_001.pdf]

Supporting Information

Curcumin-Poly(sodium 4-styrenesulfonate) Conjugates as Potent Zika Virus Entry Inhibitors

Magdalena Obłoza[1#], Aleksandra Milewska[2#] Paweł Botwina[2,3], Artur Szczepański[2], Aneta Medaj[1,4], Piotr Bonarek[5], Krzysztof Szczubiałka[1], Krzysztof Pyrć[2*], Maria Nowakowska[1*]

[1] Faculty of Chemistry, Jagiellonian University, Gronostajowa 2, 30-387 Krakow, Poland

[2] Virogenetics Laboratory of Virology, Malopolska Centre of Biotechnology, Jagiellonian University, Gronostajowa 7a, 30-387 Krakow, Poland

[3] Department of Microbiology, Faculty of Biochemistry, Biophysics and Biotechnology, Jagiellonian University, Gronostajowa 7, 30-387 Krakow, Poland

[4] Doctoral School of Exact and Natural Sciences, Jagiellonian University, Lojasiewicza 11, 30-348, Cracow, Poland

[5] Department of Physical Biochemistry, Faculty of Biochemistry, Biophysics and Biotechnology, Jagiellonian University, 30-387 Krakow, Poland

# equally contributed

Correspondence: k.pyrc@uj.edu.pl (KP); nowakows@chemia.uj.edu.pl (MN)

Keywords: Zika virus; antiviral; polymer; poly(sodium 4-styrenesulfonate); curcumin; Flavivirus

Table of Contents

Supplementary figures and tables as indicated in a paper

^{1}H NMR (600 MHz, $CDCl_3$) δ: 15.94 (bs, 1H, [h]), 7.93 (dd, J = 8.5, 1.2 Hz, 2H, [t]), 7.70 – 7.59 (m, 3H, [f, k, w]), 7.41 (t, J = 7.9 Hz, 2H, [u]), 7.19 – 7.02 (m, 5H, [b, c, l, n, o]), 6.94 (d, J = 8.2 Hz, 1H, [d]), 6.55 (d, J = 15.8 Hz, 1H, [j]), 6.49 (d, J = 15.8 Hz, 1H [g]), 5.90 (bs, 1H, [e]) 5.83 (s, 1H, [i]), 3.95 (s, 3H, [m]), 3.87 (s, 3H, [a]), 3.04 – 2.91 (m, 2H [r]), 2.84 – 2.48 (m, 2H, [p]), 2.00 (s, 3H, [s]) ppm.

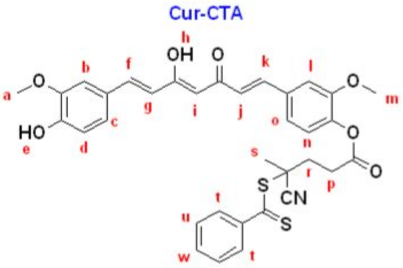

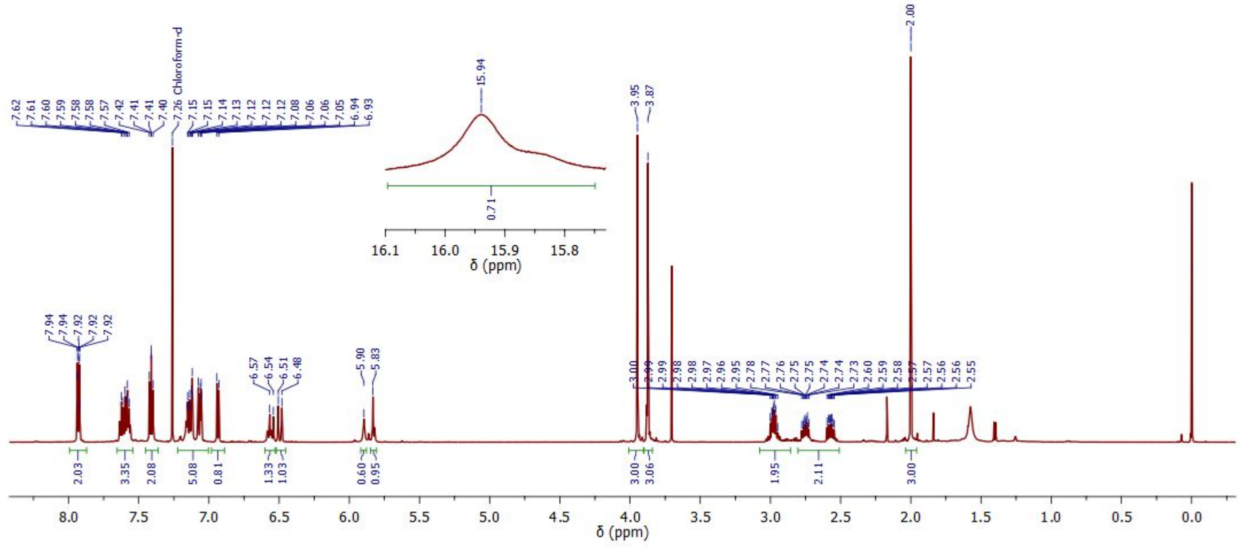

Figure S1. ^{1}H NMR spectrum of Cur-CTA in $CDCl_3$.

^{13}**C NMR** (75 MHz, $CDCl_3$) δ: 222.4, 184.7, 181.8, 169.7, 151.4, 148.2, 147.0, 144.6, 141.3, 141.0, 139.3, 134.5, 133.2, 129.3, 128.7, 127.7, 126.8, 124.6, 123.2, 122.1, 121.9, 121.1, 118.6, 115.0, 111.6, 109.8, 101.7, 56.1, 56.0, 45.9, 33.4, 31.0, 29.7, 24.3 ppm.

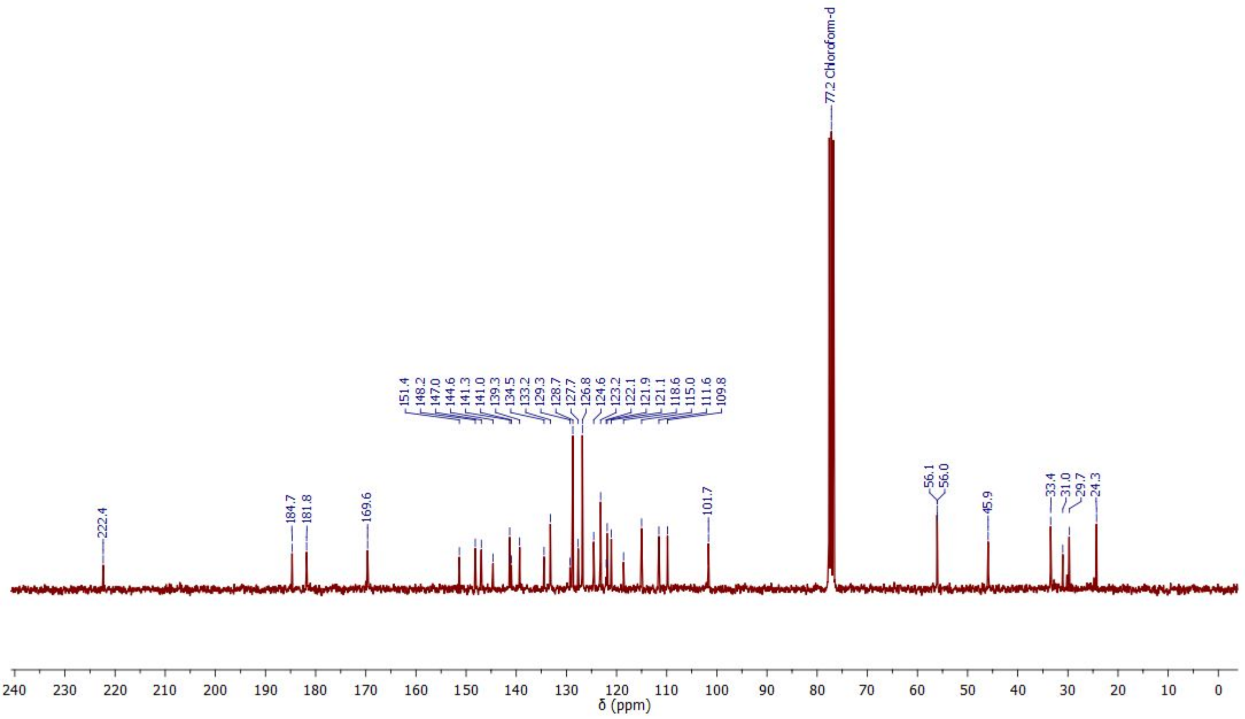

Figure S2. ^{13}C NMR spectrum of Cur-CTA in $CDCl_3$.

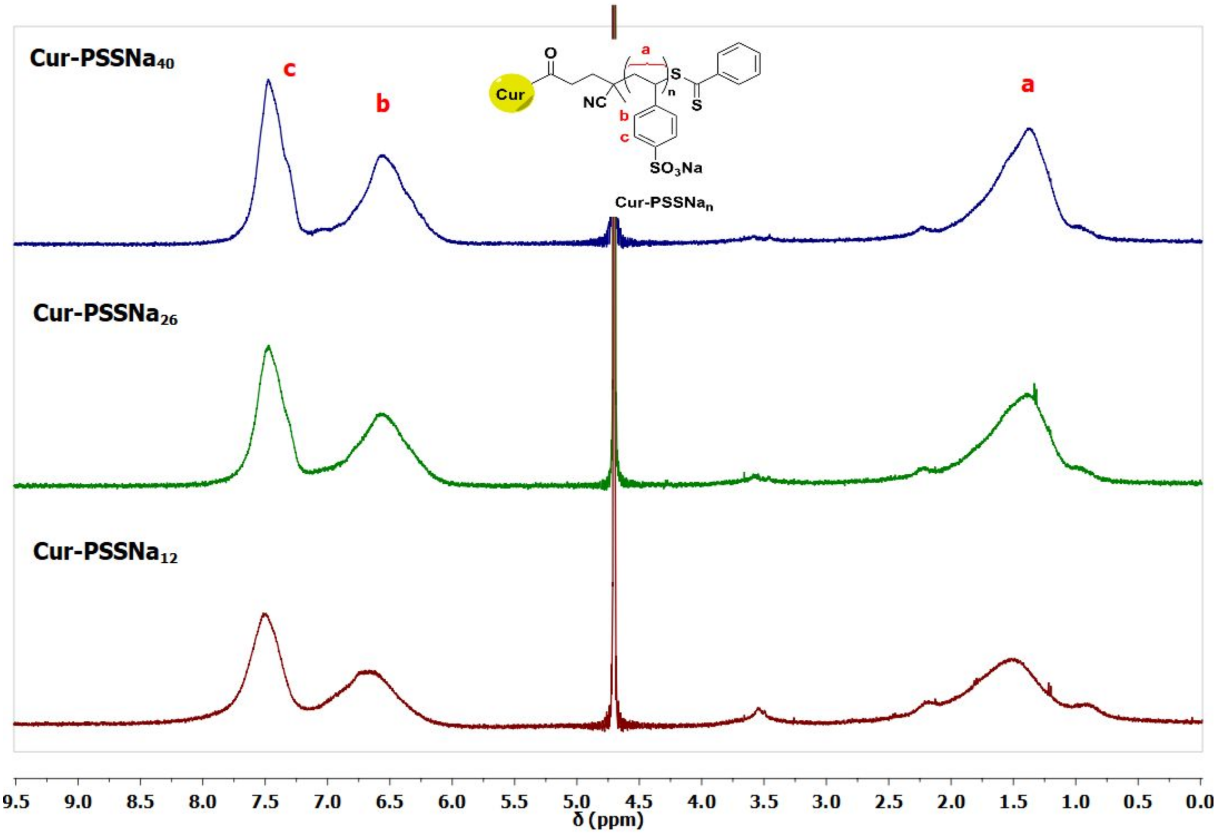

Figure S3. ^{1}H NMR spectra of Cur-PSSNa$_n$ in D_2O.

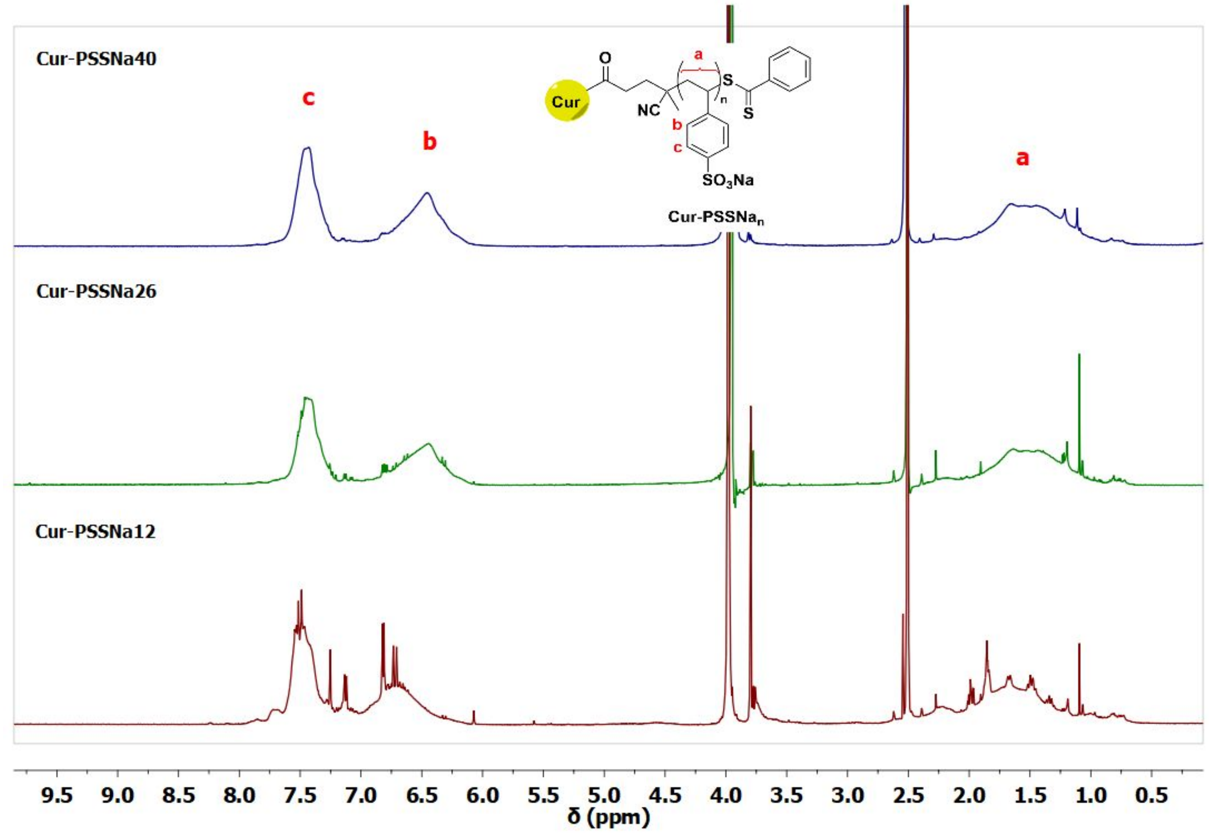

Figure S4. ^{1}H NMR spectra of Cur-PSSNa$_n$ in 20% v/v D_2O in DMSO.

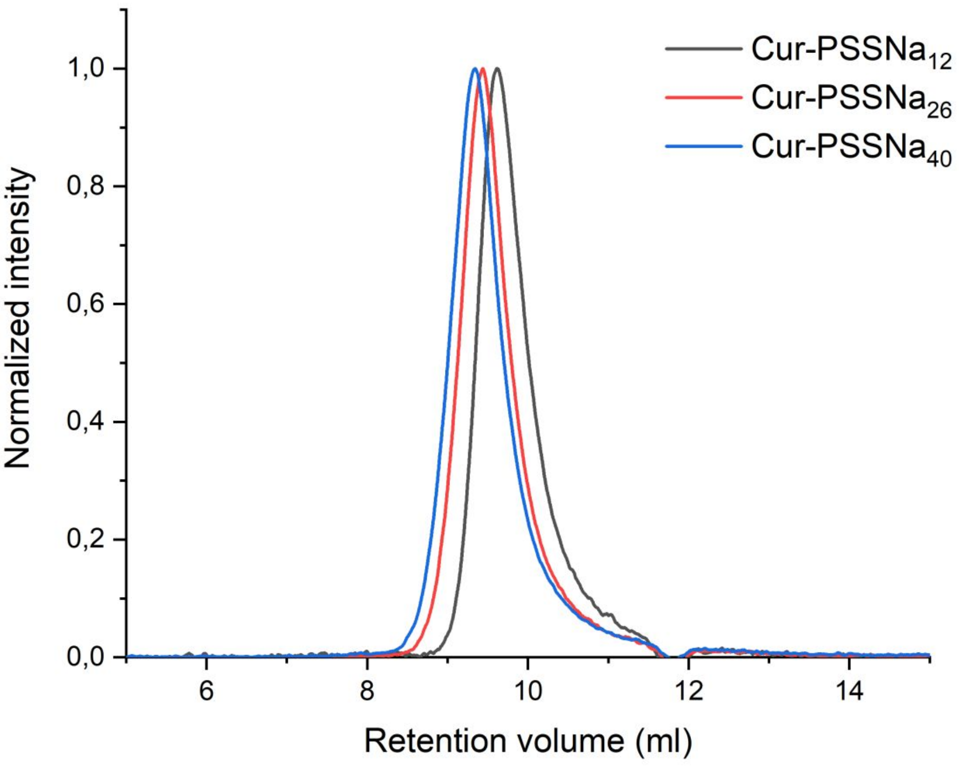

Figure S5. GPC chromatograms of Cur-PSSNa$_n$ conjugates obtained using 0.1 M NaCl aqueous solution containing 20% v/v acetonitrile as an eluent.

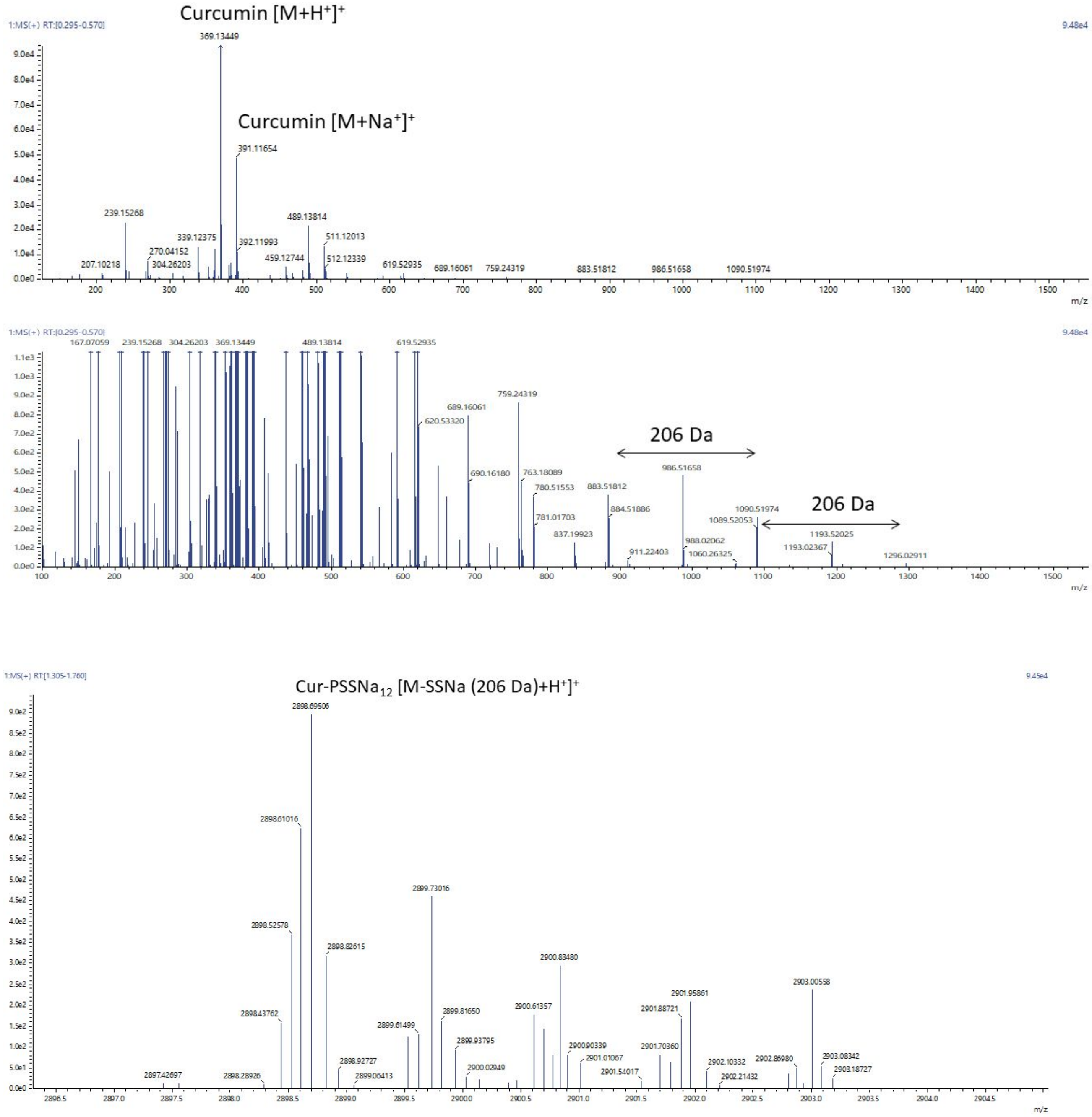

Figure S6. Positive ion ESI mass spectra of Cur-PSSNa$_{12}$ showing curcumin molecular ions (as H^+ and Na^+ adducts) in end group of conjugate (top). The plot with expanded Y axis reveals fragmentation of the polymer chain, since the differences between indicated signals correspond to the molar mass of the repeating unit, i.e., 206 Da (middle). An expanded view of the m/z 2896–2905 region is given to show the heaviest observed ions (bottom).

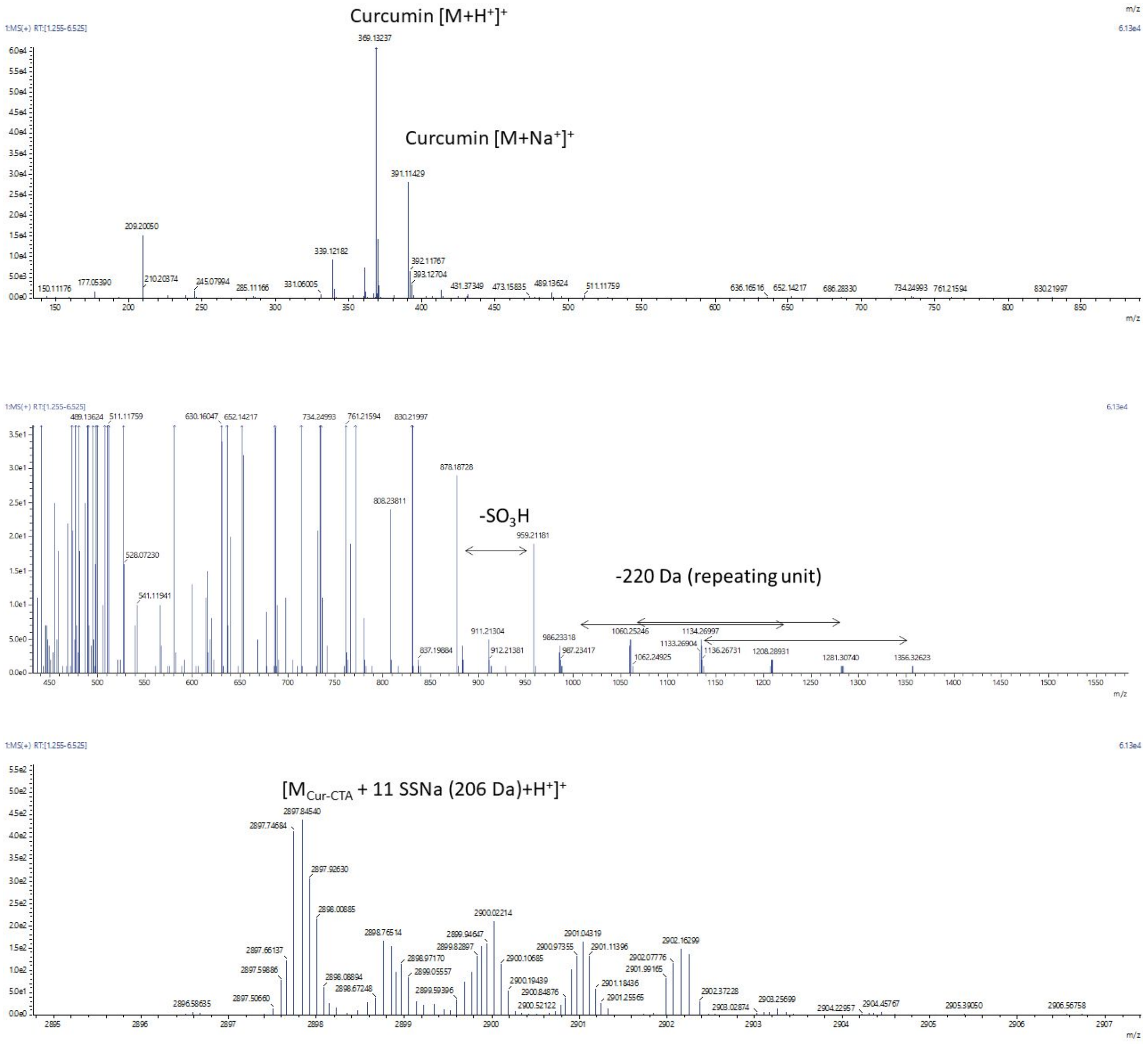

Figure S7. Positive ion ESI mass spectra of Cur-PSSNa$_{26}$ showing curcumin molecular ions as H^+ and Na^+ adducts (top). The plot with expanded Y axis reveals fragmentation of the polymer chain, since the differences between indicated signals correspond to the combined molar mass of the repeating unit and a methylene group, i.e., 220 Da (middle). An expanded view of the m/z 2896–2905 region is given to show the heaviest observed ions (bottom).

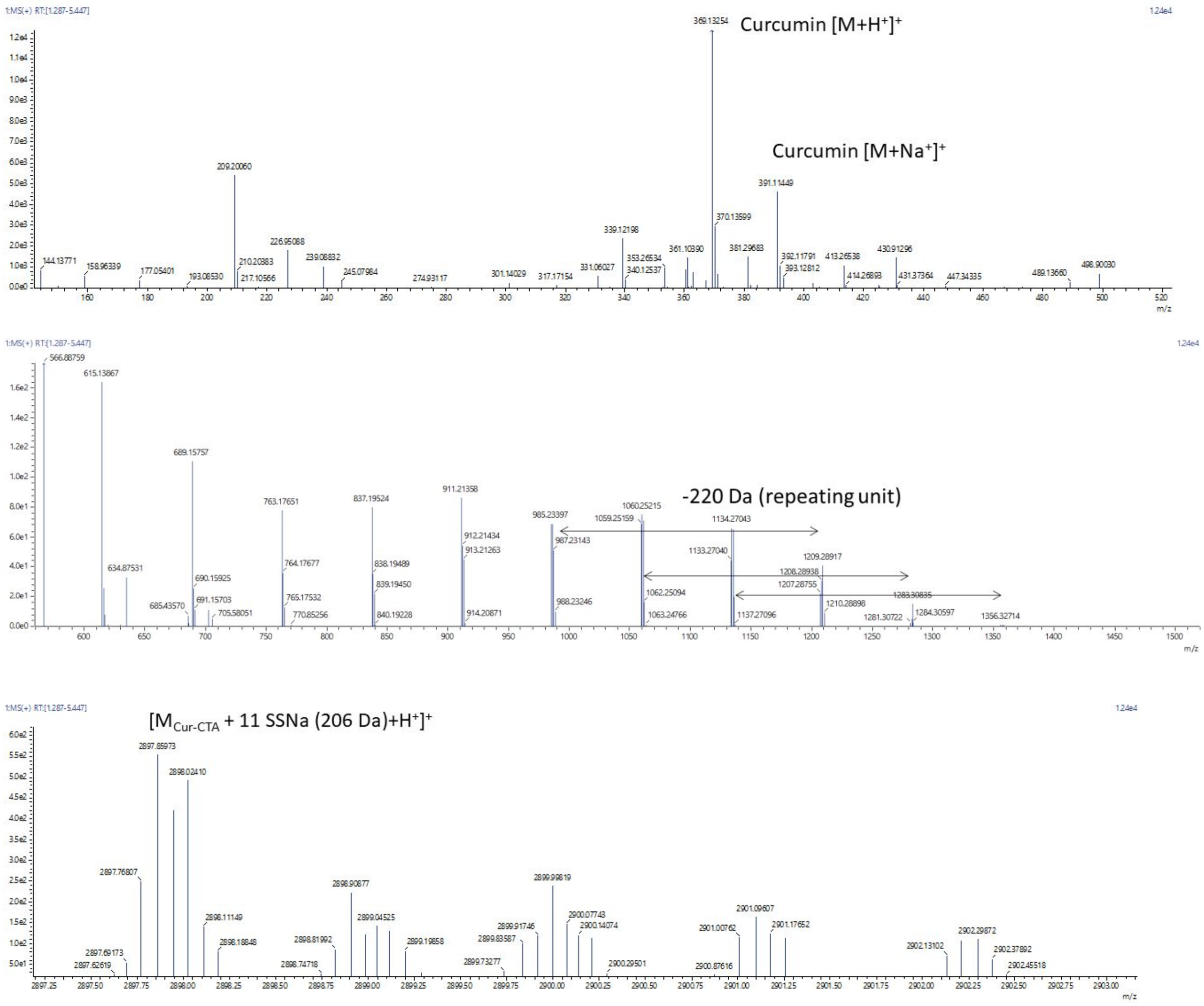

Figure S8. Positive ion ESI mass spectra of Cur-PSSNa$_{40}$ showing curcumin molecular ions (as H^+ and Na^+ adducts). The plot with expanded Y axis reveals fragmentation of the polymer chain, since the differences between indicated signals correspond to the combined molar mass of the repeating unit and a methylene group, i.e., 220 Da (middle). An expanded view of the m/z 2896–2905 region is given to show the heaviest observed ions (bottom).

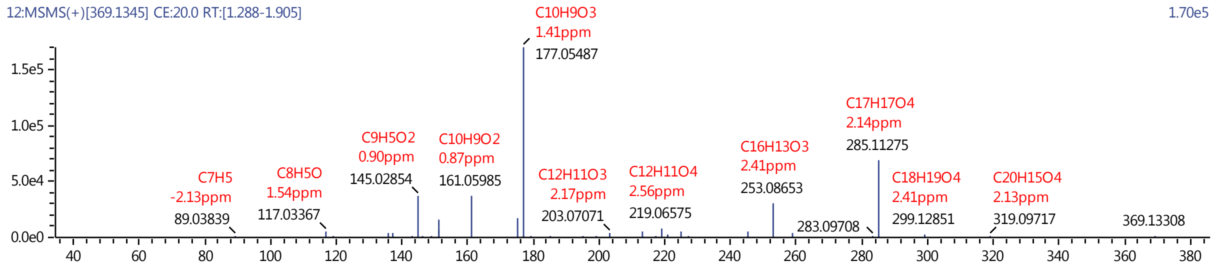

Figure S9. Positive ion ESI mass spectrum in the MS/MS mode showing fragmentation of the m/z 369 molecular ion of curcumin (collision energy 20 eV).

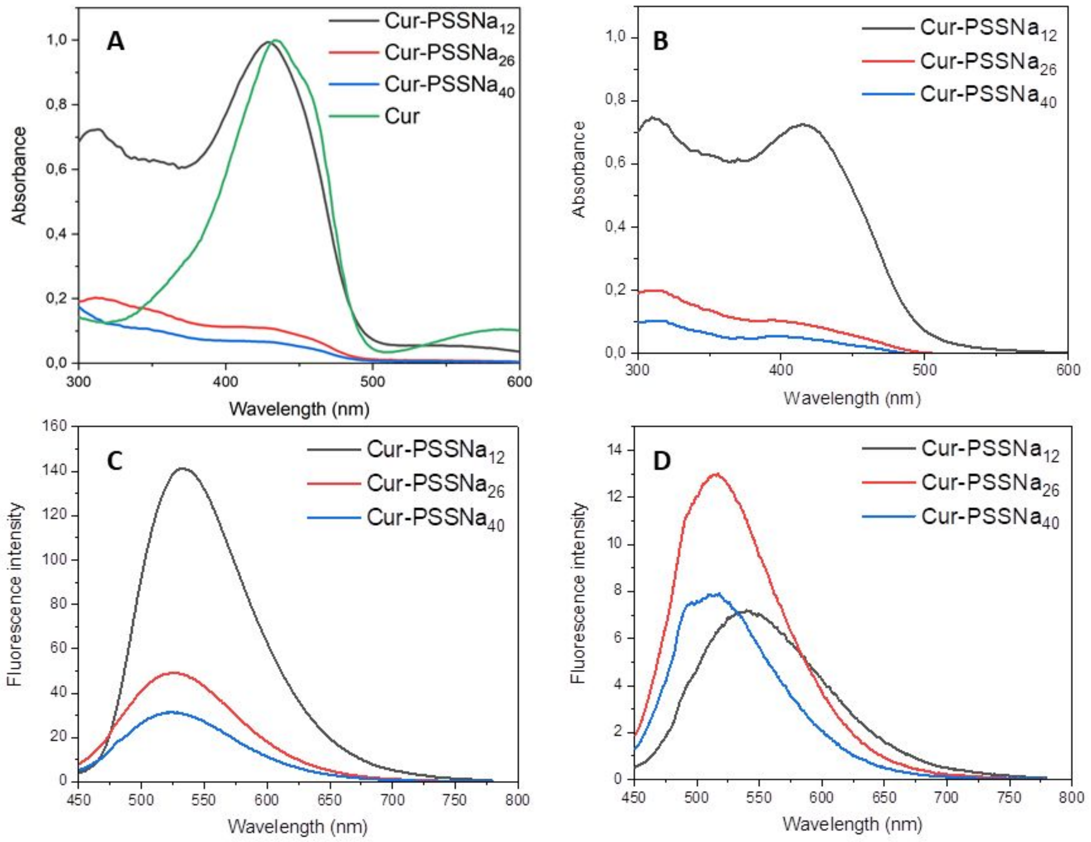

Figure S10. UV-Vis spectra of Cur–PSSNa$_n$ conjugates (0.1 mg/ml) in DMSO/PBS 4:1 v/v mixture and Cur (1.7×10^{-5} mol/dm^3) in DMSO (**A**) and in PBS (**B**). Fluorescence spectra of Cur –PSSNa$_n$ conjugates (0.1 mg/ml, λ_{ex} = 420 nm) in DMSO/PBS mixture (80/20, v/v) (**C**) and in PBS (**D**).

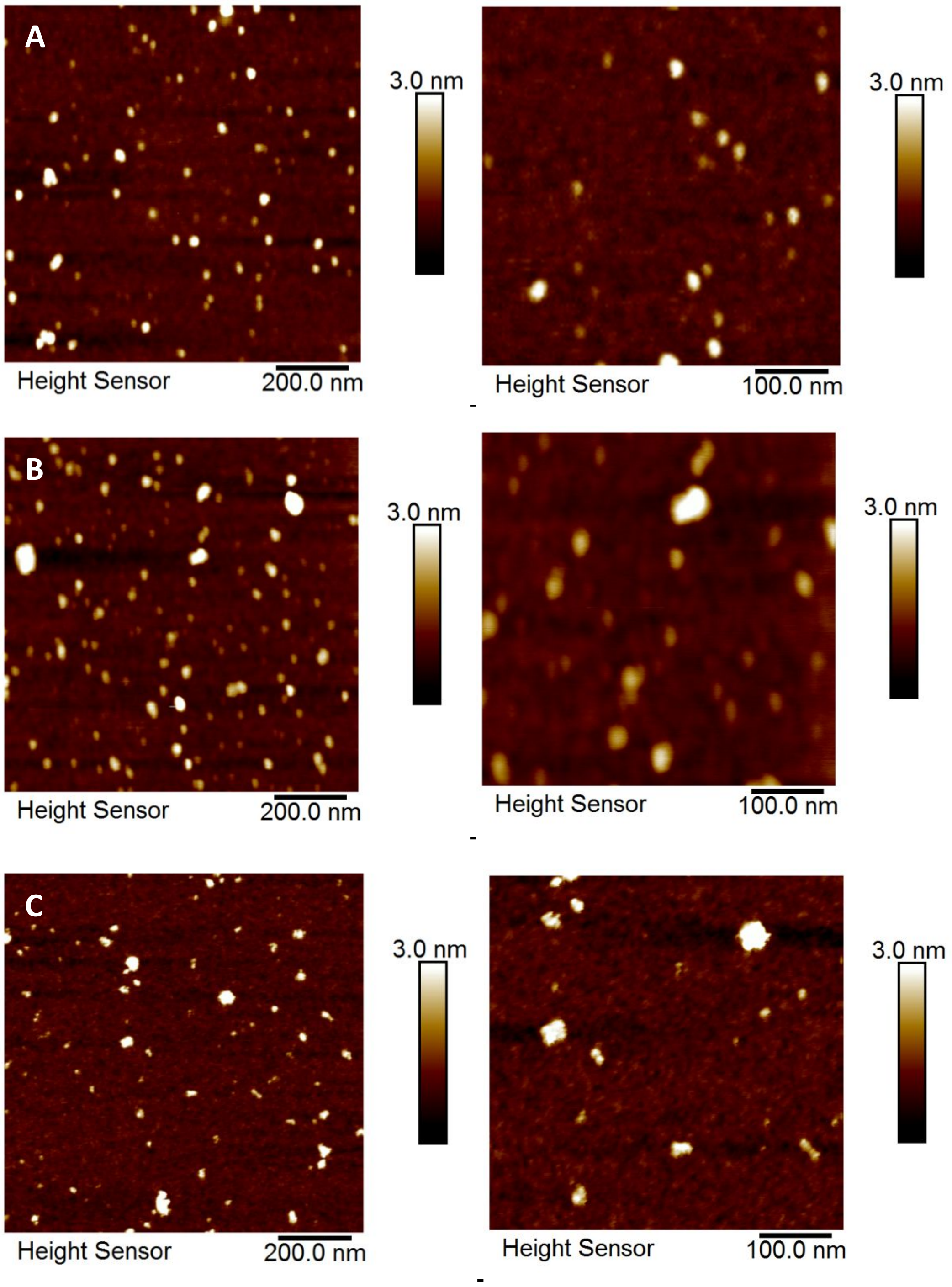

Figure S11. AFM images of Cur-PSSNa$_{12}$ (A), Cur-PSSNa$_{26}$ (B) and Cur-PSSNa$_{40}$ (C) (0.01 mg/mL in PBS).

CMC determination

The critical micelle concentration for Cur-PSSNa$_n$ was determined with fluorescence spectroscopy using diphenyl-1,3,5-hexatriene (DPH) as a fluorescence probe. Stock solutions of the fluorescent probe

DPH at a concentration of $1 \cdot 10^{-3}$ M in THF were prepared, and then diluted with PBS to obtain the final concentration of DPH equal to $5.0 \cdot 10^{-6}$ M. For the CMC determination a series of conjugate solutions were prepared at the concentrations ranging from 0.001 to 0.5 mM. The samples were incubated for 24 h in the dark at room temperature before the fluorescence was measured. The fluorescence anisotropies were determined using vertically polarized light at λ = 350 nm for excitation and both vertically (I_{VV}) and horizontally (I_{VH}) polarized light at λ = 428 nm for emission. The steady-state anisotropy (r) was calculated according to the following equation:

$$r = \frac{I_{VV} - GI_{VH}}{I_{VV} + 2GI_{VH}}$$

where: I_{VV} is defined as the intensity of light when both the excitation and emission polarizers are fixed vertically, and I_{VH} is the intensity of light when the excitation polarizer is fixed vertically and the emission polarizer is mounted horizontally; $G = I_{HV}/I_{HH}$ is an instrument correction factor (I_{HH} and I_{HV} are fluorescence intensities for horizontally fixed excitation polarizer, while the emission polarizer was fixed horizontally and vertically, respectively). CMC was determined using first derivative of fluorescence anisotropy.

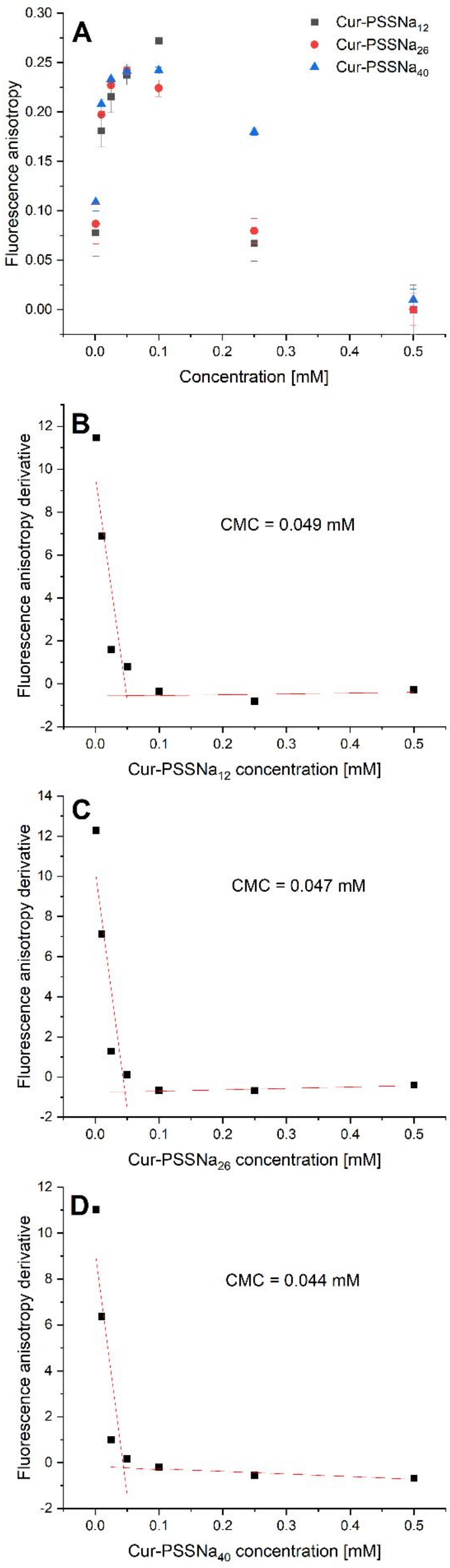

Figure S12. The fluorescence anisotropy of DPH (c = 5.0·10^{-6} M, λ_{ex} = 350 nm) as a function of conjugates concentration in PBS (A), and the first derivative of anisotropy versus the conjugate

concentration used to determine the CMC for Cur-$PSSNa_{12}$ (B), Cur-$PSSNa_{24}$ (C), and Cur-$PSSNa_{40}$ (D) conjugates.

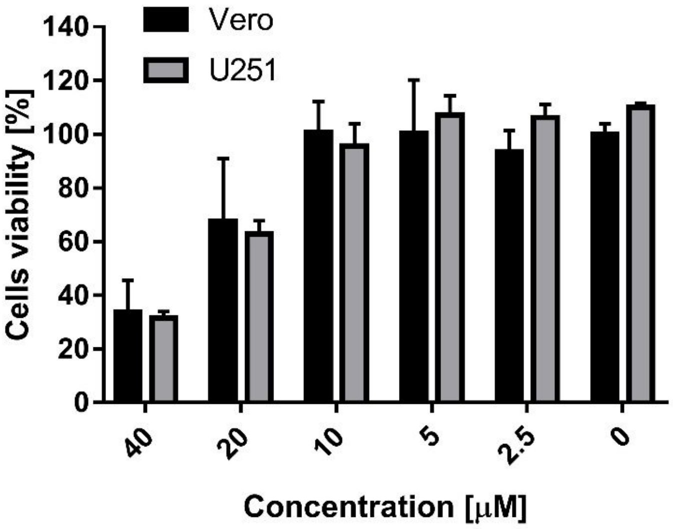

Figure S13. Cytotoxicity of Cur in Vero and U251 cells. The assay was performed in triplicate. Average values with standard deviations (error bars) are presented.

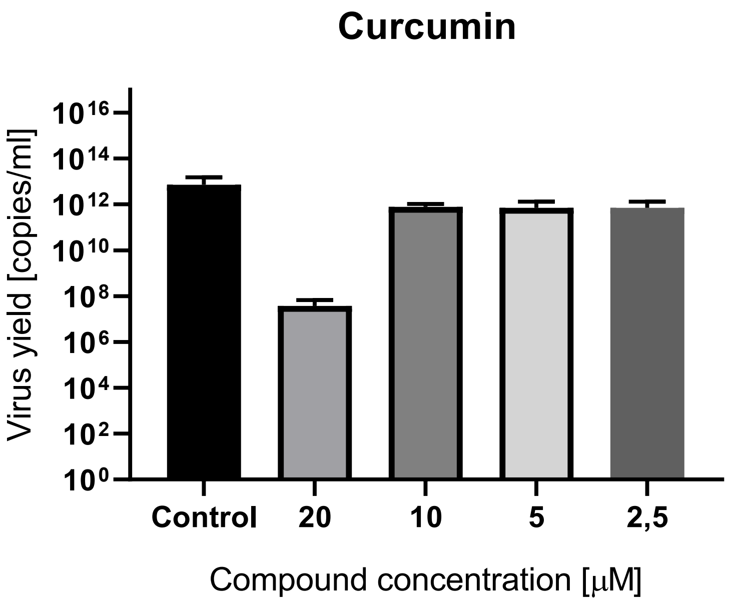

Figure S14. Inhibition of ZIKV replication by the Cur solutions at various concentrations

Table S1. Dimension, dispersity index and zeta potential of HSA-polymer aggregates and BSA-polymer aggregates in PBS determined with DLS (both protein and conjugates concentration was equal 3.3×10^{-8} mol/dm^3, T= 37°C).

**Polymer/aggregate**	**d [nm] (by volume)**	**Đ**	**Zeta potential (mV)**
**BSA**	6.46 ± 0.12	0.20 ± 0.05	- 7.8 ± 1.6
**Cur-PSSNa$_{12}$+ BSA**	7.70 ± 0.26	0.21 ± 0.03	-11.8 ± 1.3
**Cur-PSSNa$_{26}$+ BSA**	8.23 ± 0.32	0.22 ± 0.02	-14.9 ± 2.3
**Cur-PSSNa$_{40}$ + BSA**	7.96 ± 0.29	0.27 ± 0.05	-14.7 ± 1.5
**HSA**	7.44 ± 0.24	0.29 ± 0.02	- 7.5 ± 1.4
**Cur-PSSNa$_{12}$+ HSA**	8.27 ± 0.40	0.23 ± 0.07	- 8.8 ± 3.4
**Cur-PSSNa$_{26}$+ HSA**	8.59 ± 0.13	0.20 ± 0.02	-11.0 ± 0.5
**Cur-PSSNa$_{40}$ + HSA**	8.93 ± 0.75	0.38 ± 0.23	-12.5 ± 1.3
**Cur-PSSNa$_{12}$**	78.6 ± 0.40	0.20 ± 0.01	-21.1 ± 2.7
**Cur-PSSNa$_{26}$**	138.3 ± 5.35	0.23 ± 0.01	-24.0 ± 1.7
**Cur-PSSNa$_{40}$**	119.9 ± 16.1	0.29 ± 0.06	-22.7 ± 0.7

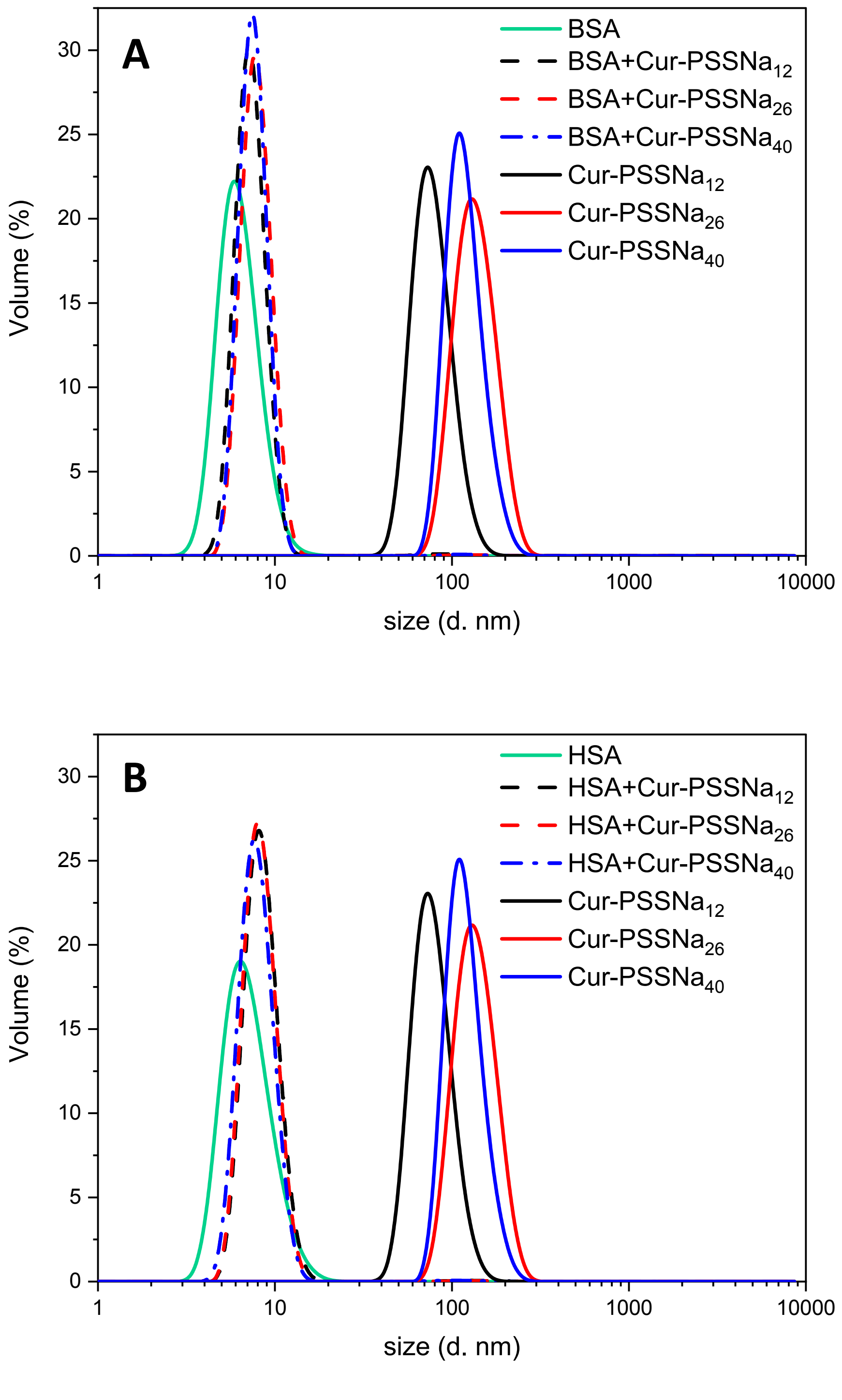

Figure S15. DLS volume-based size distribution histograms of protein – conjugate interactions in PBS at 37°C (A: with BSA – bovine serum albumin and B: with humane serum albumin). Both protein and conjugates concentration was equal $3.3 \cdot 10^{-8}$ mol/dm^3.

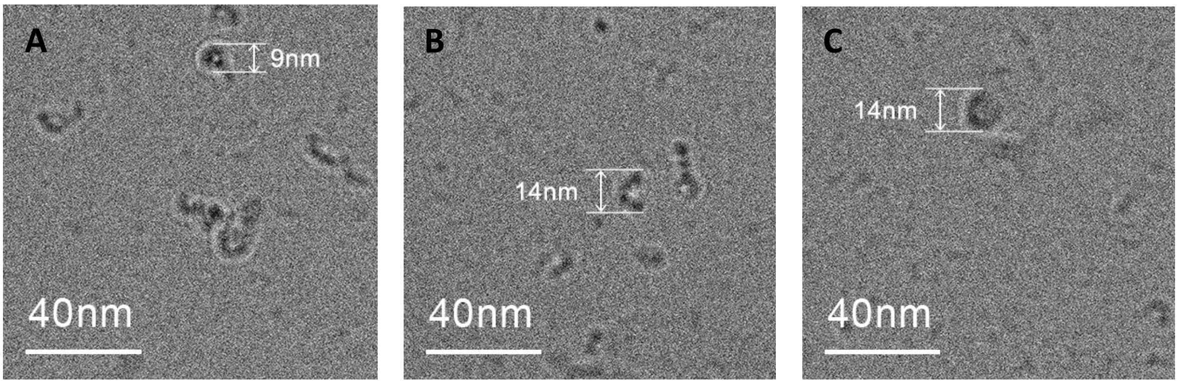

Figure S16. Cryo-TEM images of Cur-PSSNa$_{12}$ (A) Cur-PSSNa$_{26}$ (B) Cur-PSSNa$_{40}$ (C) conjugates with HSA (molar ratio 1:1, 1,5·10^{-5}M) in PBS.

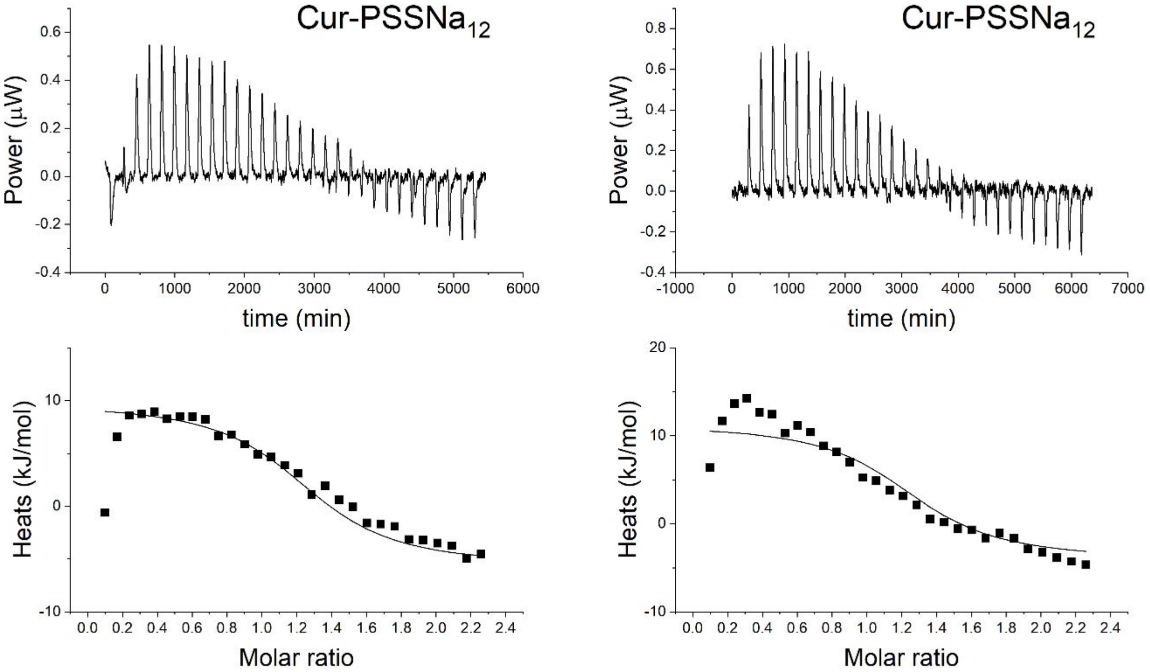

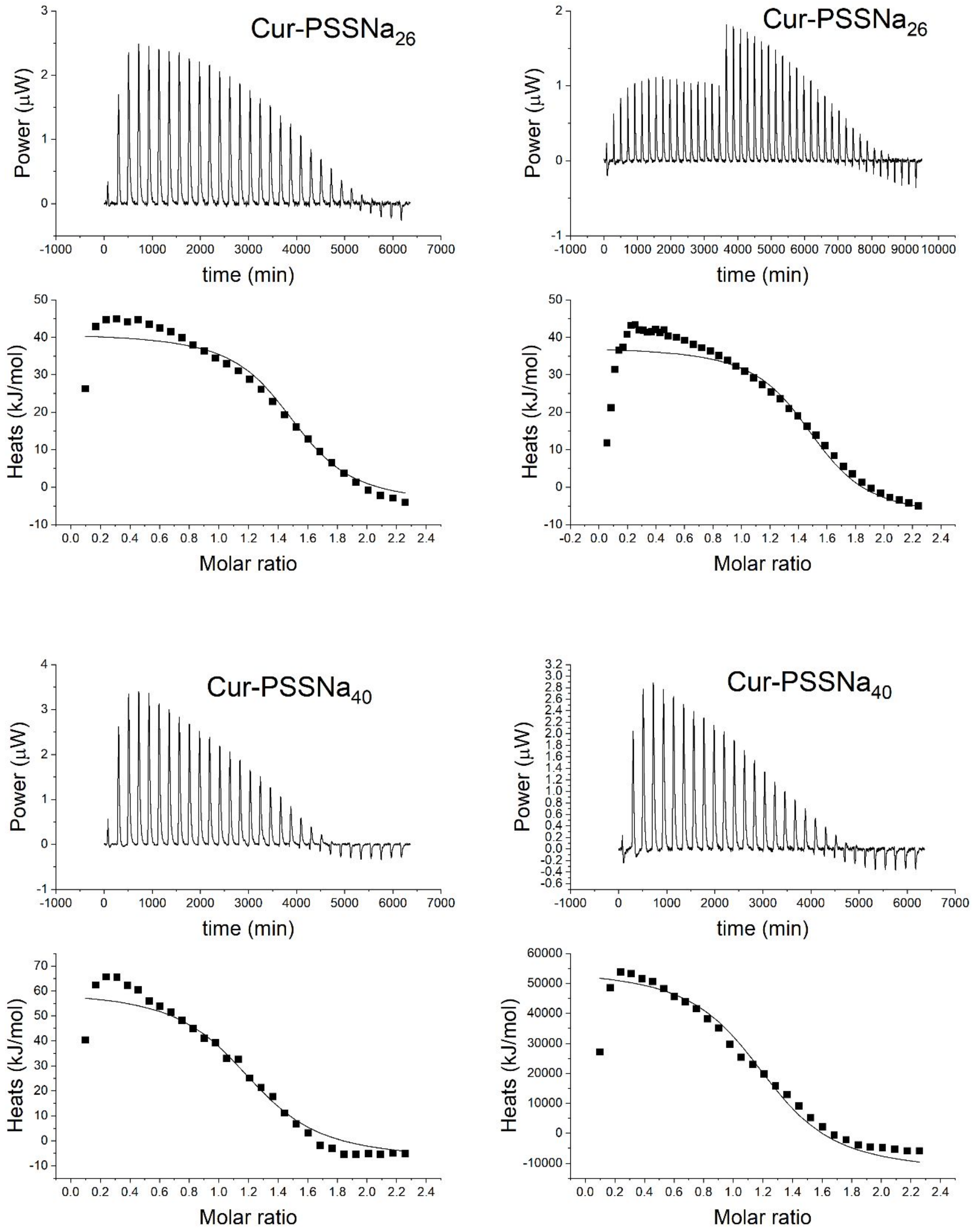

Figure S17. ITC measurements; raw data, calorimetric isotherms of the binding of Cur-PSSNa$_n$ conjugates to HSA. Experiments were carried out in PBS at 37 C. The lines represent the best fit of the one class binding sites model to the experimental data.

Literature

1. Reed, L.J.; Muench, H. A. Simple method of estimating fifty percent endpoints. *American Journal of tropical medicine and Hygiene*, 1938, 27(20), 493-497, https://doi.org/10.1093/oxfordjournals.aje.a118408

2. Ciejka. J.; Botwina, P.; Nowakowska, M.; Szczubiałka, K.; Pyrc. K. Synthetic sulfonated derivatives of poly(allylamine hydrochloride) as inhibitors of human metapneumovirus. PLoS ONE, 2019, 14(3), e 0214646, https://doi.org/10.1371/journal. pone.0214646
